# Asymmetry measures for quantification of mechanisms contributing to dynamic stability during stepping-in-place gait

**DOI:** 10.3389/fneur.2023.1145283

**Published:** 2023-04-20

**Authors:** Robert J. Peterka, Apollonia Gruber-Fox, Paige K. Heeke

**Affiliations:** ^1^Department of Veterans Affairs, National Center for Rehabilitative Auditory Research, Portland, OR, United States; ^2^Department of Neurology, Oregon Health & Science University, Portland, OR, United States

**Keywords:** balance, gait, stability, gait asymmetry, foot placement, step timing, ankle torque, stepping-in-place

## Abstract

The goal of this study is to introduce and to motivate the use of new quantitative methods to improve our understanding of mechanisms that contribute to the control of dynamic balance during gait. Dynamic balance refers to the ability to maintain a continuous, oscillating center-of-mass (CoM) motion of the body during gait even though the CoM frequently moves outside of the base of support. We focus on dynamic balance control in the frontal plane or medial–lateral (ML) direction because it is known that active, neurally-mediated control mechanisms are necessary to maintain ML stability. Mechanisms that regulate foot placement on each step and that generate corrective ankle torque during the stance phase of gait are both known to contribute to the generation of corrective actions that contribute to ML stability. Less appreciated is the potential role played by adjustments in step timing when the duration of the stance and/or swing phases of gait can be shortened or lengthened to allow torque due to gravity to act on the body CoM over a shorter or longer time to generate corrective actions. We introduce and define four asymmetry measures that provide normalized indications of the contribution of these different mechanisms to gait stability. These measures are ‘step width asymmetry’, ‘ankle torque asymmetry’, ‘stance duration asymmetry’, and ‘swing duration asymmetry’. Asymmetry values are calculated by comparing corresponding biomechanical or temporal gait parameters from adjacent steps. A time of occurrence is assigned to each asymmetry value. An indication that a mechanism is contributing to ML control is obtained by comparing asymmetry values to the ML body motion (CoM angular position and velocity) at the time points associated with the asymmetry measures. Example results are demonstrated with measures obtained during a stepping-in-place (SiP) gait performed on a stance surface that either remained fixed and level or was pseudorandomly tilted to disturb balance in the ML direction. We also demonstrate that the variability of asymmetry measures obtained from 40 individuals during unperturbed, self-paced SiP were highly correlated with corresponding coefficient of variation measures that have previously been shown to be associated with poor balance and fall risk.

## 1. Introduction

This is a methods-focused study whose goal is to introduce and to motivate the use of new quantitative measures to improve our understanding of mechanisms that contribute to dynamic balance control in the frontal plane during gait. We use the term dynamic balance to refer to the maintenance of stability during a gait task where body center-of-mass (CoM) frequently moves outside the base of support during movement (1, 2). This contrasts with static balance during stance where stability is maintained by ensuring that the CoM remains within the base of support.

While passive mechanical properties of oscillatory leg motions in the sagittal plane likely contribute to the maintenance of stable forward motion (3, 4) it is well accepted that active, neurally-mediated control is necessary to maintain dynamic balance in the frontal plane or medial-lateral (ML) direction (4–6). During successive foot placements in a walking gait, the CoM in the ML direction oscillates back and forth between the feet (2, 6, 7). To maintain dynamic balance during gait, the nervous system must execute control actions via mechanisms that ensure a stable oscillating pattern of ML body motion (8, 9).

Justification for focusing on ML balance includes evidence that ML balance during gait is disturbed to a much greater extent by ML visual motion stimuli than is AP balance by AP stimuli suggesting a dominant contribution of active mechanisms for stabilizing ML balance compared to AP balance during gait (4). Additional justification comes from studies in older adults demonstrating greater difficulty with ML than AP balance (10–14) and tasks challenging ML balance were better predictors of falls in older subjects than tasks challenging AP balance (15).

This study illustrates new measures for characterizing dynamic balance control by making use of data collected using a stepping-in-place (SiP) paradigm. The primary use of a SiP paradigm has been to document the effect of vestibular dysfunction on heading direction (16). Similarly, Agathos et al. (17) used SiP to investigate the influence of optic flow on self-motion perception. There is only a small literature that has used SiP to investigate the mechanics of gait. Brenière (18, 19) considered that the pattern of ML CoM and CoP motions during SiP closely resembled those that occur during a walking gait such that experimental results from SiP could be used to draw conclusions about “the relationships between body parameters and gravity and the central programming of locomotor parameters.” Garcia et al. (20) made comparisons of stepping parameters obtained using both SiP and forward walking in healthy adults and adults with hemiplegia. The similar results from both paradigms led to the overall conclusion that the results “may provide solid evidence that stepping-in-place and gait are inherently related.” We made use of the similarity in ML motion in SiP and walking gait and the need for dynamic balance control in both gaits to develop new measures that can characterize the contributions of mechanisms that control dynamic balance.

There are multiple mechanisms that can potentially contribute to ML dynamic balance control. Many studies considered that a mechanism based on “step-width regulation” is the primary mechanism for controlling dynamic balance (5, 21–29) with a theoretical study showing that step-width regulation is an efficient mechanism for controlling ML balance (5). Step-width regulation involves the subject placing a foot further from or closer to the body’s midline on each step, to generate a gravity-induced corrective torque that is appropriate to maintain dynamic balance (with the corrective torque proportional to the distance between the body CoM and the stance foot position, and with sequential foot placements determined in relation to sensory-detected deviations from the desired body movement).

Ankle torque and reaction torque (latter from the coordinated motion of the upper body relative to the lower body and also called counter-rotation mechanism) have also been recognized as contributors to ML balance control in humans (7, 21, 30–37). Additionally, variations in actions affecting mainly forward walking progression can also contribute to ML control to the extent that there is crosstalk between sagittal and frontal control mechanisms. These include modulation of push-off torque (4, 8, 35, 38) and alteration in the direction of travel (steering control) (39).

Largely unrecognized in the physiological literature is the potential contribution of step timing mechanisms for controlling ML dynamic balance. In studies of balance in cats (40) and humans (21, 41), researchers found that changes in single-leg support time were observed in response to sudden ML body displacements caused by impulsive perturbations. Additionally, a recent study identified that an ML margin of stability measure, assumed to be indicative of the quality of balance control, was found to be closely related to adjustments in single-leg support times during various treadmill walking manipulations while the modulation of step width showed less co-variation (42). Significantly, in a robotics study of gait control, in both model-simulations and actual implementations in a cat-sized quadruped robot, researchers developed an algorithm that relied entirely on leg loading and unloading to alter step timing to control ML dynamic balance during forward walking (43, 44) and, thus, demonstrated the ability of this mechanism alone to achieve ML dynamic balance control.

The potential for step-timing to contribute to ML dynamic balance control can be understood by considering the physics of body motion during the single-leg stance phase of a gait cycle. At the beginning of each single-leg stance phase the body CoM is moving toward the stance leg, but the CoM is medial to the stance foot (7). The location of the body CoM relative to the foot provides a gravity-induced frontal plane component of torque that initially slows, then stops, and finally reverses the ML progression of the CoM. By extending/shortening the duration of the single-leg stance phase, torque acts over a longer/shorter time interval, imparting greater/lesser corrective action to control motion in the ML direction and facilitating stability of ML dynamic balance. Furthermore, the swing phase duration for each leg, the time from toe off to the next heel contact, could potentially be separately regulated to alter the duration over which ML corrective torque is available during the previous or subsequent stance phases.

To quantify the relationship between deviations of ML CoM motion from the normal step cycle to the modulation of step parameters contributing to dynamic balance control, we developed four unitless asymmetry measures that compared normalized step-to-step changes in step parameters. We refer to these measures as Step Width Asymmetry (SWA), Ankle Torque Asymmetry (ATA), Stance Duration Asymmetry (StDA), and Swing Duration Asymmetry (SwDA). All of these asymmetry calculations were formulated with the property that their signs are indicative of step-to-step changes that contribute to compensation for directional deviations in ML CoM body motion from a stable ML gait cycle. In this methods-focused paper we use results from SiP tests to illustrate the calculation of these four asymmetry measures which are applicable to the analysis of both SiP and forward walking gaits. For forward walking gait, the potential exists that asymmetry measures that characterize step-to-step changes in push-off torque (4, 8, 35, 38) and steering control (39) could be developed.

While studies evaluating unperturbed variations in CoM motion in relation to compensatory actions (45, 46) have contributed to understanding balance control during gait, the application of ML balance perturbing stimuli have also been used to provide evidence of control actions that contribute to balance corrections. Perturbing stimuli have included physically moving the subject by pushing or pulling (41, 47–49), application of galvanic stimulation (34, 36, 50), perturbing the visual environment (35, 51–54), and moving both the visual scene and walking surface (55). We demonstrate the application of our asymmetry measures in both unperturbed and perturbed conditions.

In unperturbed conditions the variability of asymmetry time series would likely be related to traditional variability measures (i.e., coefficient of variation of step width, step time, swing time, stride time). Since traditional gait variability measures have been linked to gait stability deficits and falls (56–58), the demonstration of a high correlation between asymmetry variability and traditional gait variability measures would suggest that asymmetry variability could provide a similar indicator of gait deficits. Furthermore, a demonstration that asymmetry measures are associated with mechanisms that correct for deviations of ML CoM motion could additionally link traditional gait variability measures with mechanisms regulating dynamic balance control.

The purpose of this paper is to define the algorithms to calculate these new asymmetry measures, to show how biomechanical measures can be processed in order to relate both stimulus-evoked and spontaneous variation in CoM motion to the asymmetry measures, and to give examples of these methods. Additionally, we show that the variability of asymmetry measures calculated in conditions where no stimulus perturbation is applied are highly correlated with step variability measures that have previously been shown to relate to the quality of balance control during gait and to disability (56, 57, 59–62). Application of our new measures may provide a bridge between studies using differing methodologies (with and without perturbations, with and without special subject populations) by offering a method which can easily be employed to facilitate understanding mechanisms contributing to ML dynamic balance control in a variety of settings and in different patient populations.

## 2. Materials and methods

### 2.1. Participants

Example data were drawn from healthy participants who were all Veterans with no reported balance deficits. Participants included a ‘young’ age group (*N* = 20, age range 25–43 years, mean 32 years, 16 male) and an ‘old’ age group (*N* = 20, age range 65–82 years, mean 72 years, 19 male). All participants gave written informed consent in the study whose recruitment procedures and experimental protocols were approved by the Veterans Affairs Portland Health Care System Institutional Review Board.

Subjects were screened using a questionnaire to rule out current and past conditions that could be contributors to balance dysfunction (neurological deficits, concussion, numbness, heart disease, fainting, arthritis and joint pain, motion limitations, diabetes, meningitis, sensory dysfunction including hearing, vision, vestibular/balance disorders) and to query their ability to stand and walk for 20 min. VA medical records were accessed to verify responses to the subject questionnaire and to identify whether subjects were taking medications that could affect balance. Subjects were not included if disorders were present, fitness was low, or medications affecting balance were used.

### 2.2. Data collection, protocols, and gait measures

Tests were performed on a custom balance platform (see Supplementary Figure S1) consisting of dual force plates (each 25 × 50 cm) that measured vertical forces from each foot at the corners of each force plate. Subjects stood or stepped-in-place with one foot on each force plate and faced a semicircular visual surround with a high contrast scene. The platform and visual-surround could tilt side-to-side to perturb ML balance with tilt angles determined by servo-controlled motors. The rotation axes of platform and visual surround were perpendicular to the subject’s frontal plane, located at ankle joint height, and aligned with the middle of the platform. A custom real-time LabVIEW data collection program generated stimuli and collected experimental data (LabVIEW version 2015; NI PXI-8115 controller; NI PXI 6259 multifunction module, National Instruments, Austin, TX, United States)^1^. The program generated (1) the waveform used for the surface-tilt or visual-tilt stimulus and, on some trials, (2) a metronome pacing signal. The program recorded (1) vertical forces from eight force transducers (MLP-150 load cells, Transducer Techniques, Temecula, CA)^2^ located near the corners of each force plate, (2) signals from four optical distance sensors (Sharp Corp., Sakai, Osaka, Japan)^3^ used to measure ML foot locations on the force plate surfaces, (3) signals from two potentiometers (model CP-2UT, Midori America Corp., Irvine, CA, United States)^4^ used to measure ML body motion at hip and shoulder levels, (4) measures of actual surface-tilt or visual-tilt angles, and (5) the metronome pacing signal. The sampling rate for all signals was 200/s.

Surface-tilt and visual-tilt stimuli were defined by a waveform based on a 5-stage pseudorandom ternary maximal length sequence [PRTS; (63)] that was mathematically integrated and scaled to give 36.3-s duration stimulus profiles with peak-to-peak tilt amplitudes of 0° (fixed surface or visual tilt angle), 1°, 3°, or 6°. Six continuous PRTS cycles were presented per trial. The ML angular tilt of the stance surface was controlled by a servomotor (Model 4102DCM000B1CCF006, Cleveland Motion Controls, Billerica, MA)^5^ with custom gear reduction using Amacoil/Uhing linear drive nuts (Amacoil, Inc., Aston, PA, United States)^6^ and controller (Model BL20-40, BL Linear Amplifier, Aerotech Inc., Pittsburgh, PA, United States)^7^. The visual-surround was controlled by the same model servomotor, also with custom gear reduction, and controller (Model SD2-720–40-1 amplifier, Servo Dynamics Corp., Chatsworth, CA, United States)^8^. The metronome rate (~92.56 beats per minute) was chosen to give 56 steps per 36.3-s PRTS cycle duration.

Subjects wore a harness with attachments to overhead beams to prevent falls with the attachment straps adjusted so they did not provide support during testing. Tests were performed without shoes or socks to ensure availability of tactile information (see next paragraph). Headphones were used to mask ambient sounds and to deliver metronome beats to control step timing on most trials. On trials without metronome pacing, audio book recordings were played to maintain subject alertness.

T-shaped foam strips (6 mm height) were taped to the platform surface with the vertical portion of the T (9.5 cm width) located between the feet, and the horizontal portion of the T (8 cm width) located at the front of the platform. Subjects were instructed to maintain toe contact on each step with the horizontal foam strip to minimize forward or backward drift during SiP. The vertical foam strip provided feedback to reduce drift in the ML direction. The width of the vertical foam strip was less than the distance between the feet during SiP such that subjects typically did not make contact with the strip during SiP. Subjects were instructed to be aware that if they did make contact with the vertical strip then they were to move laterally on subsequent steps to avoid continued contact with the vertical strip.

Each subject performed 27 tests with each test having a duration of 258 s: 11 were SiP tests performed with metronome pacing, 11 were stance tests, and 5 were SiP tests performed without metronome pacing. The 11 SiP tests with metronome and the 11 stance tests both included tests in 3 conditions (visual tilt, surface tilt with eyes open and closed) each with 1°, 3°, or 6° stimulus amplitudes. There were two additional eyes open and closed tests on a fixed and level surface. The non-metronome-paced SiP tests included tests performed eye open and closed on a fixed surface and 3 eyes closed tests performed with 1°, 3°, or 6° surface-tilt stimuli. All tests were performed in randomized order in 2 or 3 sessions on separate days. Five-minute rest periods were provided every 4^th^ test or as needed. Subjects were given practice trials with metronome pacing prior to testing. Not every metronome-paced test was performed with perfect compliance throughout each test, but perfect compliance was not necessary for the calculation of our asymmetry measures.

#### 2.2.1. Foot placement measures

Two optical distance sensors were placed on the outer edges of the platform (Sharp Corp. model GPY0A41SK0F with 4–30 cm range) to measure the distance from the platform edge to the outer edge of each foot (at approximate location of the ankle joint). Two additional sensors (Sharp Corp. GPY0A51SK0F with 2–15 cm range) were placed between the feet to measure the distance from the midpoint of the platform to the inner edges of each foot. Data from these sensors were used to calculate the ML foot center of each foot during the stance phase of SiP gait and later used to calculate the ‘step width’ defined as the ML distance between right and left foot centers at time points that were at the approximate midpoint of the double support phase of the gait cycle.

#### 2.2.2. Gait timing measures

Recordings of the time courses of total vertical force under each foot were used to calculate foot-on and foot-off contact times for each foot and each step following the methods related to those described by Hausdorff et al. (64). Briefly, an initial force threshold, whose value was about 1% of total body weight, was used to identify approximate foot-on contact times (when the force first rose above the threshold) and foot-off contact times (when the force first fell below the threshold). The contact times were refined by calculating the rate-of-change of the force signals (force velocity) and searching in the vicinity of the previously identified contact times for the time point where the force velocity fell below a velocity threshold value that was close to zero. The identified foot-on and foot-off contact times were used to calculate the duration of each foot’s contact with the surface (i.e., stance duration) and the duration when each foot was not in contact with the surface (i.e., swing duration).

#### 2.2.3. Vertical force and center-of-pressure (CoP) measures

Measures from the force plate transducers were used to calculate the total vertical force under each foot, the ML CoP displacement under each foot when the foot was in contact with the force plate, and the whole-body ML CoP displacement.

#### 2.2.4. Medial–lateral body motion measures

Medial–lateral body displacements at hip and shoulder heights were recorded using ‘sway rods’ that consisted of Earth-fixed potentiometers (Midori America Corp.) to which were attached the sway rods (aluminum arrow shafts) with the distal ends of the rods placed in hooks attached to the subject a hip and shoulder levels. The potentiometer voltages were recorded and processed, taking into account trigonometric relationships, to calculate ML displacements. At the beginning of test sessions subjects performed a calibration trial where they were instructed to sway very slowly side-to-side using a variety of upper and lower body orientations. The calibration data were analyzed to determine the linear regression factors that defined the relationship between ML hip and shoulder displacements and the ML center-of-mass (CoM) displacement relative to the center of the platform using the assumption that the CoP displacement will be vertically aligned with CoM displacement for very slow motions [see (65)]. However, because a subject did not remain perfectly centered on the platform during SiP, adjustments were made to the measured ML hip and shoulder displacements so that these displacements represented displacements relative to the subject’s ‘path’ defined as the trajectory over time of the ML location of a point midway between the feet (see next section). The regression factors from the calibration trial were then applied to the path-corrected hip and shoulder displacement to calculate the CoM displacement relative to the path. Finally, using the path-corrected CoM displacement and an estimate of a subject’s CoM height above the ankle joint based on body segment measures (66), the subject’s ML CoM tilt angle was calculated. This ML CoM tilt angle was the primary output variable used in later stages of analysis.

#### 2.2.5. Medial–lateral path analysis

**Figure 1:**
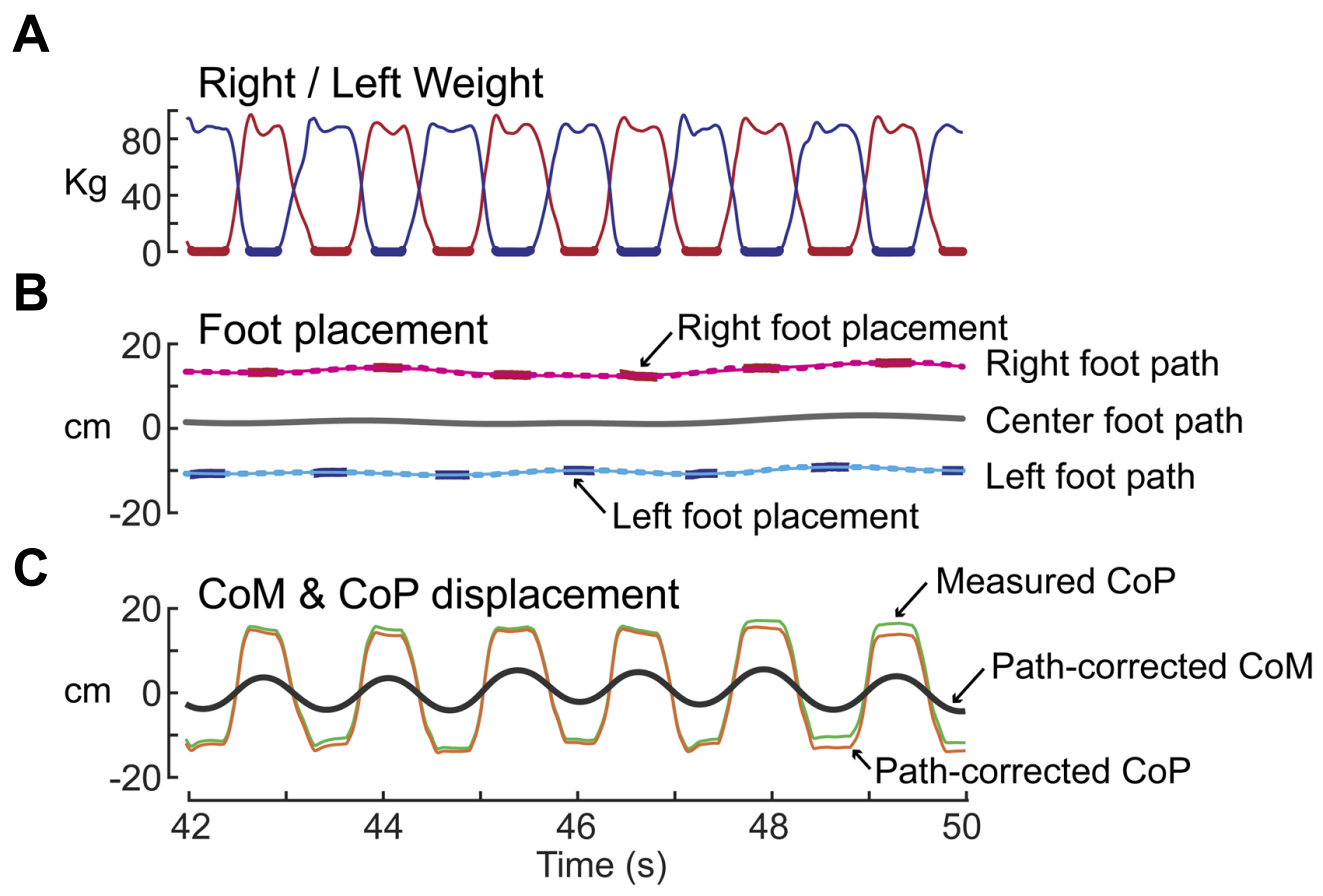
Path analysis of stepping-in-place data for the purpose of estimating the trajectory of the midpoint between the right and left foot locations over time. **(A)** Shows time courses of weight changes measured by the right (red) and left (blue) force plates. Thick lines indicate single-leg stance segments of gait. **(B)** Shows the processing of right and left foot placement data (red and blue bars) to calculate the right and left foot paths (thin solid lines) and then averaging of these paths to calculate the center foot path (thick black line). **(C)** The center foot path was used to derive path-corrected measures of the whole-body medial-lateral center-of-pressure (CoP) and center-of-mass (CoM) displacements.

The path trajectory was estimated using the procedures illustrated in Figure 1. The path trajectory was used to compensate for any potential ML drift during SiP, allowing CoM displacements to be referenced to the ML location of the calculated path which defined the time-varying location midway between the feet throughout the test. The vertical force measures from the R and L force plates were used to detect the time segments when only one foot was in contact with the surface (Figure 1A, thick bars). During these foot-contact segments the optical distance sensors provided measures used to calculate the ML location of the foot center for each foot to give a set of discontinuous ML foot locations (Figure 1B, thick bars). Separately for each foot, a moving window average with window width 1.5 s was applied to the discontinuous foot location segments. The averaging was applied to all ML foot displacement values during contact segments that were within the 1.5 s window as the window center point incremented through time resulting in a continuous time series that ramped toward each successive foot location (Figure 1B, dotted lines). A phaseless lowpass filter (4 order 0.5 Hz cutoff using Matlab filtfilt function; The MathWorks, Inc., Natick, MA; Matlab, 2021a) was then applied to the windowed time series for each foot (Figure 1B, thin solid lines) to calculate the trajectory of the ML displacement of each foot. Then the R and L foot trajectories were averaged to give the overall path trajectory (Figure 1B, thick solid line). Finally, the computed path trajectory was used to provide a path-corrected CoP and CoM displacement trajectories (Figure 1C) and the path-corrected CoM displacement was used to compute the CoM tilt angle about an origin defined by the path and located at ankle height.

We chose to quantify the dynamic state of the body using the angular tilt of the CoM. Asymmetry measures could have been calculated using the horizontal CoM displacement relative to the path with similar results. An advantage of using CoM angle is that this provides a normalization that accounts for subjects with different CoM heights. It is important to recognize that our CoM angular measure represents the state of the body orientation relative to Earth vertical and is not a measure proportional to the gravitational torque exerted on the body during the single leg stance phases of gait which depends on the distance between the CoM and foot position.

### 2.3. Gait asymmetry measures

Four gait asymmetry measures were defined by comparing gait measures across adjacent pairs of steps over the course of an experimental trial.

#### 2.3.1. Step width asymmetry

**Figure 2:**
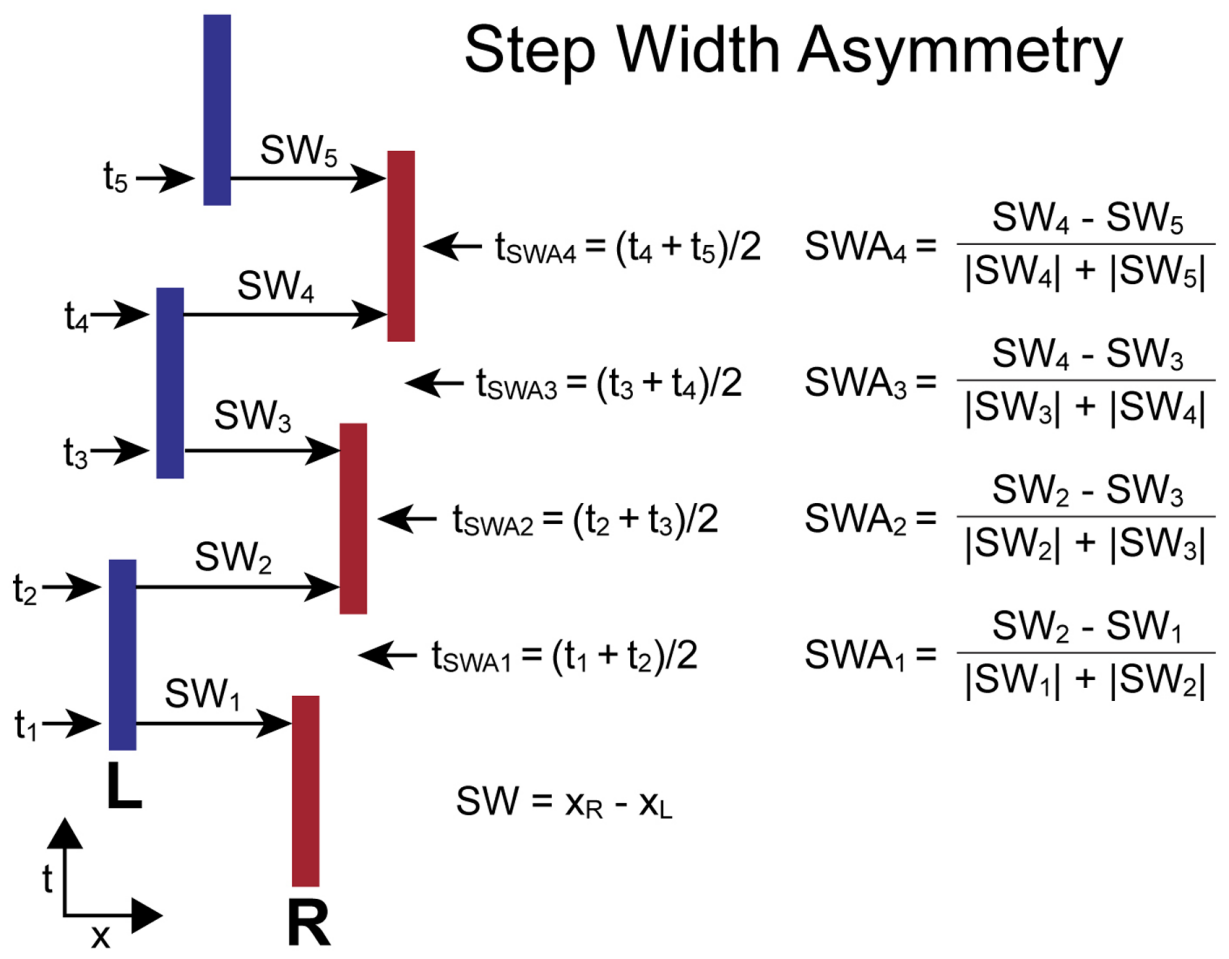
Step Width Asymmetry (*SWA*) calculation parameters and formula. Times *t_i_* are the times at the midpoints of the double-support phases at which the step widths, *SW_i_*, are measured. The *SWA* measures are based on a normalized comparison of adjacent pairs of *SW* measures. The time point, *t_SWAi_*, associated with each *SWA* measure is the average of the mid-double-support times used for the *SWA* calculation. The calculations are shown for the condition where the first step is with the right (R) foot and differs from the calculation when the first step is with the left (L) foot so that positive *SWA* measures are always indicative of a movement toward the R independent of whether the first step was with the R or L foot.

The *SWA* measure compares adjacent pairs of ML (frontal plane) step widths (*SW_i_*, *SW_i + 1_*) calculated from foot placement measures. Specifically, *SW* is the ML coordinate of the right-foot center location minus the ML coordinate of the adjacent left-foot center location. The *SWA* is normalized by the sum of the absolute values of the two step widths to give a unitless measure indicating the relative change in step width and the direction of lateral motion resulting from the combination of two adjacent steps. The equation for calculating *SWA* depends on which foot stepped first. When the first step is by the right (R) foot (as in Figure 2), the equations are:

$SWA_{i}=\left(SW_{i+1}-SW_{i}\right)/\left(\left|SW_{i}\right|+\left|SW_{i+1}\right|\right)$ for *i* = odd (i.e., R-to-L steps)
$SWA_{i}=\left(SW_{i}-SW_{i+1}\right)/\left(\left|SW_{i}\right|+\left|SW_{i+1}\right|\right)\;\;$ for *i* = even (i.e., L-to-R steps)

When the first step is by the left (L) foot, the equations are:

$SWA_{i}=\left(SW_{i}-SW_{i+1}\right)/\left(\left|SW_{i}\right|+\left|SW_{i+1}\right|\right)\;$ for *i* = odd (i.e., L-to-R steps)
$SWA_{i}=\left(SW_{i+1}-SW_{i}\right)/\left(\left|SW_{i}\right|+\left|SW_{i+1}\right|\right)\;$ for *i* = even (i.e., R-to-L steps)

For both calculations the R-to-L step width is subtracted from the adjacent L-to-R step width. Therefore, the *SWA* asymmetry measure has a positive value when the adjacent steps produced a net movement to the R, and a negative value when the net movement is to the left. Individual *SW* values can be negative in the case of crossover steps.

The time (*t_i_*) assigned to the *i*th step width measure (*SW_i_*) is defined as the time at the approximate midpoint of the double-support phase of the gait cycle which was calculated by taking the mean of the time points associated with adjacent R and L stance periods. The time assigned to the *i*th *SWA* measure (*t_SWAi_*) is defined as the average of the two adjacent *t_i_* times used in the *SWA* calculation. Thus, *t_SWAi_* is approximately at the midpoint of the single-leg stance phase of the gait cycle.

Figure 2 illustrates the calculation of *SWA* for a stepping pattern that was continuously progressing to the R and began with a R step. In this case, all *SW* measures with even indexes are L-to-R steps, and these steps are larger than all *SW* measures with odd indexes (R-to-L steps). The *SWA_i_* measures will have positive signs for all indexes. The assignment of a time to each *SWA* measure allows for the comparison of body motion variables measured at the times associated with the *SWA* measures.

#### 2.3.2. Ankle torque asymmetry

The *ATA* measure compares values related to ankle torque (*AT_i_*, *AT_i + 1_*) measured at the midpoint of single leg stance phases of adjacent R and L steps. An ankle everting or inverting torque produces a ML shift in the location of the CoP under a foot. This CoP shift moves the effective point of force application either closer to or farther away from the body CoM causing a decrease or increase, respectively, in the net torque that affects the ML acceleration of the body. The *AT* for each foot is calculated by subtracting the recorded ML foot placement (*FP* – a measure of the ML location of the foot center) from the ML *CoP* value at the midpoint of the single leg stance phase (*AT_i_* = *CoP_i_* – *FP_i_*). The *ATA* is normalized by dividing by foot width (*FW*). The equation for calculating *ATA*, which does not depend on which foot stepped first, is:


$$ATA_{i}=\frac{\left(AT_{i}+AT_{i+1}\right)}{FW}.$$

The time (*t_i_*) assigned to the *i*th *AT* measure (*AT_i_*) is defined as the time at the midpoint of single-leg contact time with the surface. The time assigned to the *i*th *ATA* measure (*t_ATAi_*) is defined as the average of the two adjacent *t_i_* times used in the *ATA* calculation. Thus, *t_ATAi_* is approximately at the midpoint of the double-leg stance phase of the gait cycle.

**Figure 3:**
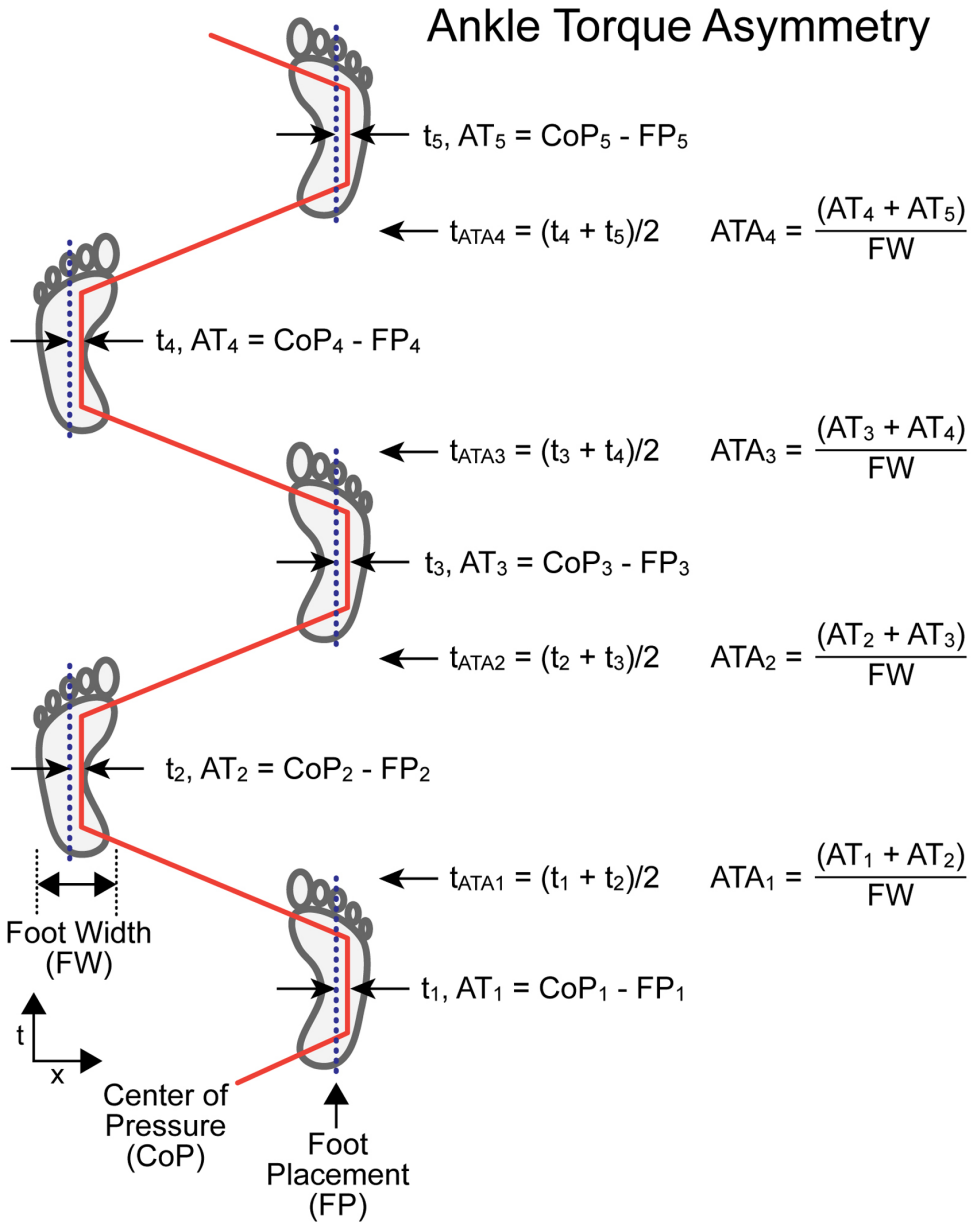
Ankle Torque Asymmetry (*ATA*) calculation parameters and formula. Times *t_i_* are the times at the midpoints of the single-leg support phases at which the lateral distance between the center-of-pressure under the foot (*CoP_i_*) and the center line of the foot placement (*FP_i_*) were used to calculate a value proportional to ankle torque (*AT_i_*) but with units of displacement (e.g., cm). The *AT* measures from adjacent steps were added and then divided by the foot width (*FW*) to give a unitless *ATA* measure associated with the paired steps at a time, t*_ATAi_*, that occurs at approximately the mid-point of the double-leg support phase. The example shows a condition where the CoP under both the right and left feet during the single-leg support phases are always displaced to the right relative to the foot center.

Figure 3 illustrates the calculation of *ATA* for a stepping pattern where the CoP is shifted toward the right relative to the foot placement for both R and L feet. In this example, the rightward shift in the R foot is caused by ankle inversion and in the L foot is caused by ankle eversion. Thus, *AT* values for both R and L feet have positive values and *ATA* measures at all indexes have positive values. This pattern provides a corrective torque appropriate to compensate for a rightward bias in body lean during gait. The assignment of a time to each *ATA* measure allows for the comparison of body motion variables measured at the times associated with the *ATA* measures.

#### 2.3.3. Stance duration asymmetry

The *StDA* measure compares adjacent R and L leg stance durations (*StD_i_*, *StD_i + 1_*). *StD* for each foot is the duration from the time of first contact with the surface (typically at heel-contact during walking but usually at ball of foot contact during SiP) to the time the foot leaves the surface (typically at toe-off during walking). The *StDA* is normalized by the sum of the two stance durations used in its calculation to give a unitless measure indicating the relative change in duration of stance phases and the leg that had the longest contact duration. The equation for calculating *StDA* depends on which foot stepped first. When the first step is by the R foot, the equations are:


$$StDA_{i}=\frac{\left(StD_{i}-StD_{i+1}\right)}{\left(StD_{i}+StD_{i+1}\right)}\mathrm{for}\;i=\mathrm{odd}.$$

$$StDA_{i}=\frac{\left(StD_{i+1}-StD_{i}\right)}{\left(StD_{i}+StD_{i+1}\right)}\mathrm{for}\;i=\mathrm{even}.$$

When the first step is by the L foot, the equations are:


$$StDA_{i}=\frac{\left(StD_{i+1}-StD_{i}\right)}{\left(StD_{i}+StD_{i+1}\right)}\mathrm{for}\;i=\mathrm{odd}.$$

$$StDA_{i}=\frac{\left(StD_{i}-StD_{i+1}\right)}{\left(StD_{i}+StD_{i+1}\right)}\mathrm{for}\;i=\mathrm{even}.$$

For both calculations the L foot stance duration is subtracted from the adjacent R foot stance duration. Therefore, the *StDA* asymmetry measure has a positive value when the R foot remained on the surface longer than the L foot on adjacent steps, and a negative value when the L foot remained on the surface longer than the R foot.

The time (*t_i_*) assigned to the *i*th stance duration measure (*StD_i_*) is defined as the time at the midpoint of a foot’s contact time with the surface. The time assigned to the *i^th^ StDA* measure (*t_StDAi_*) is defined as the average of the two adjacent *t_i_* times used in the *StDA* calculation. Thus, *t_StDAi_* is approximately at the midpoint of the double-leg stance phase of the gait cycle.

**Figure 4:**
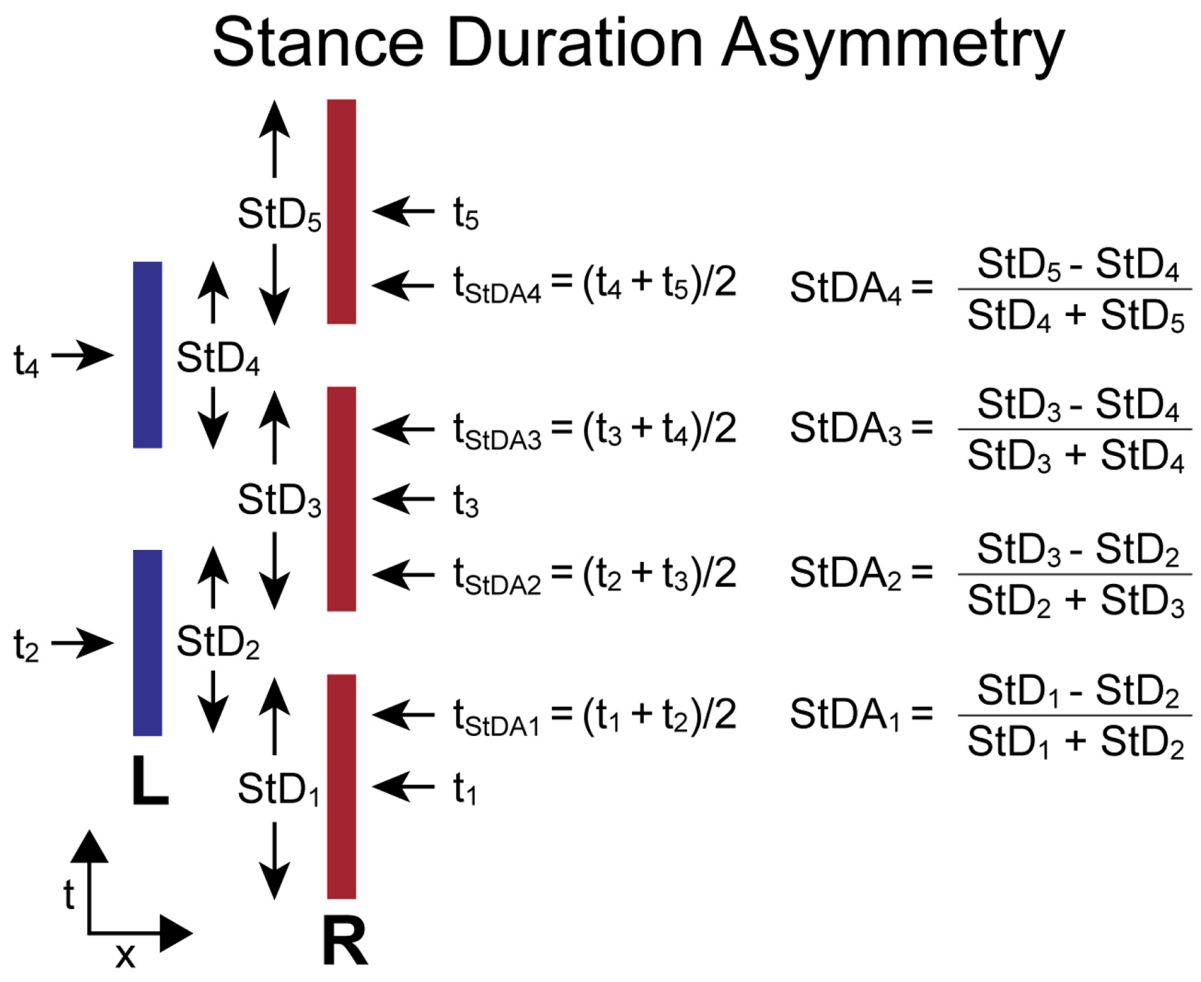
Stance Duration Asymmetry (*StDA*) calculation parameters and formula. Times *t_i_* are the times at the midpoint in time when a foot is in contact with the surface defined as the stance durations (*StD_i_*). The difference between the values of *StD* on adjacent steps divided by their sum determines the value of *StDA*. The time point (*t_StDAi_*) associated with each *StDA* measure is the average of the midpoints in time of the right and left stance phases used for the *StDA* calculation and occurs approximately at the midpoint of the double-leg support phase of gait. The calculations are shown for the condition where the first step is with the right (R) foot and differs from the calculation when the first step is with the left (L) foot so that a positive *StDA* measure is always indicative of a pattern of stepping where the R foot contact time is greater than the L foot contact time.

Figure 4 illustrates the calculation of *StDA* for a stepping pattern that began with a R step. All R foot stance durations are slightly longer than L foot stance durations. Thus, the *StDA* measures at all indexes will have positive values. The assignment of a time to each *StDA* measure allows for the comparison of body motion variables measured at the times associated with the *StDA* measures.

#### 2.3.4. Swing duration asymmetry

The *SwDA* measure compares adjacent R and L leg swing durations (*SwD_i_*, *SwD_i + 1_*). *SwD* for each foot is the duration from the time when the foot leaves the surface (typically toe-off time during walking) to the following time of foot contact with the surface (typically the heel-contact time during walking). The *SwDA* is normalized by the sum of the two swing durations used in its calculation to give a unitless measure indicating the relative change in swing-leg duration and the leg that had the longest swing duration. The equation for calculating *SwDA* depends on which foot stepped first. When the first step is by the R foot, the equations are:


$$SwDA_{i}=\frac{\left(SwD_{i}-SwD_{i+1}\right)}{\left(SwD_{i}+SwD_{i+1}\right)}\mathrm{for}i=\mathrm{even}.$$

When the first step is by the L foot, the equations are:


$$SwDA_{i}=\frac{\left(SwD_{i}-SwD_{i+1}\right)}{\left(SwD_{i}+SwD_{i+1}\right)}\mathrm{for}i=\mathrm{odd},$$

$$SwDA_{i}=\frac{\left(SwD_{i+1}-SwD_{i}\right)}{\left(SwD_{i}+SwD_{i+1}\right)}\mathrm{for}i=\mathrm{even}.$$

For both calculations the R foot swing duration is subtracted from the adjacent L foot swing duration. Therefore, the *SwDA* asymmetry measure has a positive value when the R foot swing duration is shorter than the L foot swing duration on adjacent steps, and a negative value when the R foot swing duration is longer than the L foot swing duration.

The time (*t_i_*) assigned to the *i*th swing duration measure (*SwD_i_*) is defined as the time at the midpoint of a leg’s swing phase. The time assigned to the *i*th *SwDA* measure (*t_SwDAi_*) is defined as the average of the two adjacent *t_i_* times used in the *SwDA* calculation. Thus, *t_SwDAi_* is approximately at the midpoint of the double-leg stance phase of the gait cycle.

**Figure 5:**
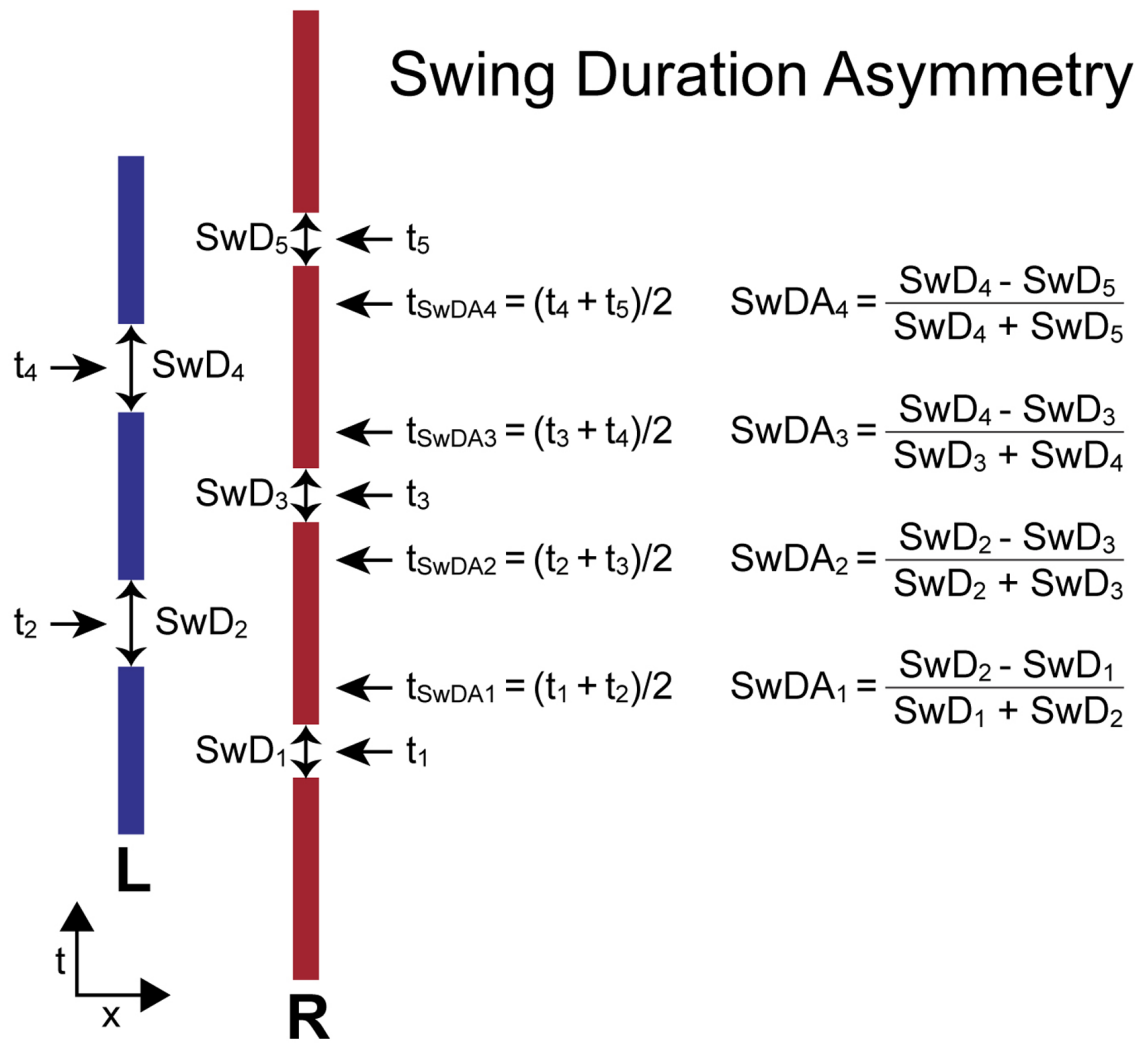
Swing Duration Asymmetry (*SwDA*) parameters and formula. Times *t_i_* are the times at the midpoint in time of the swing phase of gait when a foot is not in contact with the surface defined as the swing durations (*SwD_i_*). The difference between the values of *SwD* on adjacent steps divided by their sum determines the value of *SwDA*. The time point (*t_SwDAi_*) associated with each *SwDA* measure is the average of the midpoints in time of the right and left swing phases used for the *SwDA* calculation and occurs approximately at the midpoint of the double-leg support phase of gait. The calculations are shown for the condition where the first step is with the right (R) foot and differs from the calculation when the first step is with the left (L) foot so that a positive *StDA* measure is always indicative of a pattern of stepping where the R foot *SwD* is shorter than the L foot *SwD*.

Figure 5 illustrates the calculation of *SwDA* for a stepping pattern that began with a R step. All R foot swing durations are slightly shorter than L foot swing durations. Thus, the *SwDA* measures at all indexes will have positive values. The assignment of a time to each *SwDA* measure allows for the comparison of body motion variables measured at the times associated with the *SwDA* measures.

### 2.4. Relating asymmetry measures to CoM deviation

To the extent that modulations of step width, ankle torque, and stance and swing durations contributed to the maintenance of dynamic balance we anticipated the asymmetry measures would be correlated with deviations from the normal symmetric oscillatory pattern of ML CoM angular motion about earth-vertical expected during gait on a level surface. CoM angular deviations from the normal pattern can occur due to normal step-to-step variability caused by imperfect control of gait and from application of stimuli that either directly evoke body sway (an external push) or stimuli that indirectly, via sensory integration mechanisms, bias the mean body orientation away from an oscillatory pattern about earth vertical.

To measure the CoM deviation from upright we applied a phaseless 4-order Butterworth lowpass filter with a cutoff frequency of 0.5 Hz to the measured CoM motion. This cutoff frequency was below the metronome-paced stepping frequency of about 0.77 Hz and thus eliminated most of the oscillatory sway pattern while preserving the longer-term deviations of CoM from upright. The effects of different choices of the cutoff frequency were investigated (see Section 3.3).

A central difference calculation applied to the filtered CoM was used to obtain a measure of CoM velocity. We refer to the filtered CoM and CoM velocity time series as deviation signals ΔCoM and ΔCoM_vel_, respectively. The values of ΔCoM and ΔCoM_vel_ were sampled at the corresponding asymmetry time points and then regression analysis was applied to explore the relationship between body motion and asymmetry:

*Asym* = *offset* + *P* * ΔCoM + *V* * ΔCoM_vel_.

Where *P* is the regression position factor, *V* the regression velocity factor, *offset* a constant component, ΔCoM is the deviation from upright orientation, and ΔCoM_vel_ is the deviation from zero velocity. An *R*^2^ value was calculated for each regression to measure the amount of variance of the data accounted for by the regression equation. The *P* and *V* factors quantify the extent to which step width, ankle torque, stance duration, and swing duration asymmetry measures were modulated by CoM motion. This method was applicable on trials with or without external stimuli and with or without metronome pacing. On metronome-paced trials it was not necessary that the subject remained fully in sync with the metronome across the entire trial.

When there was no evidence of habituation or adaptation across a trial, cycle-to-cycle variability of the ΔCoM and ΔCoM_vel_ signals and the asymmetry measures can be reduced by averaging across stimulus cycles. For the across-stimulus-cycle averaging of asymmetry measures, each 36.3-s duration stimulus cycle was divided into 56 bins (2 bins per gait cycle for metronome-paced trials). Asymmetry measures that fell within each bin and the time of occurrences of those asymmetry measures were accumulated across all the stimulus cycles. Then the asymmetry values within each bin and their times of occurrence were averaged and these cycle-averaged data were used in the regression analysis. The reduced variability from cycle averaging provided clearer visualization of the regression results and comparisons of the predicted to the measured asymmetry values on trials where ML sway was evoked by external stimulation. The cycle-averaged comparisons were applicable on trials whether or not the subject was able to maintain perfect metronome-paced stepping and on non-metronome paced trials.

To explore how asymmetry variability related to traditional gait variability measures, the variability of asymmetry measures, expressed as the standard deviation (SD) of the step-to-step asymmetry measures obtained from non-metronome paced SiP tests with no applied stimulus were compared with coefficient of variation (CV) measures of step width, stance time, and swing time measures.

## 3. Results

We first present data to demonstrate the various data processing steps involved in constructing and analyzing our asymmetry variables. Data from non-perturbed trials are then presented with comparisons made between the variability of our asymmetry measures and conventional gait variability measures utilized in the current literature.

### 3.1. Example results

**Figure 6:**
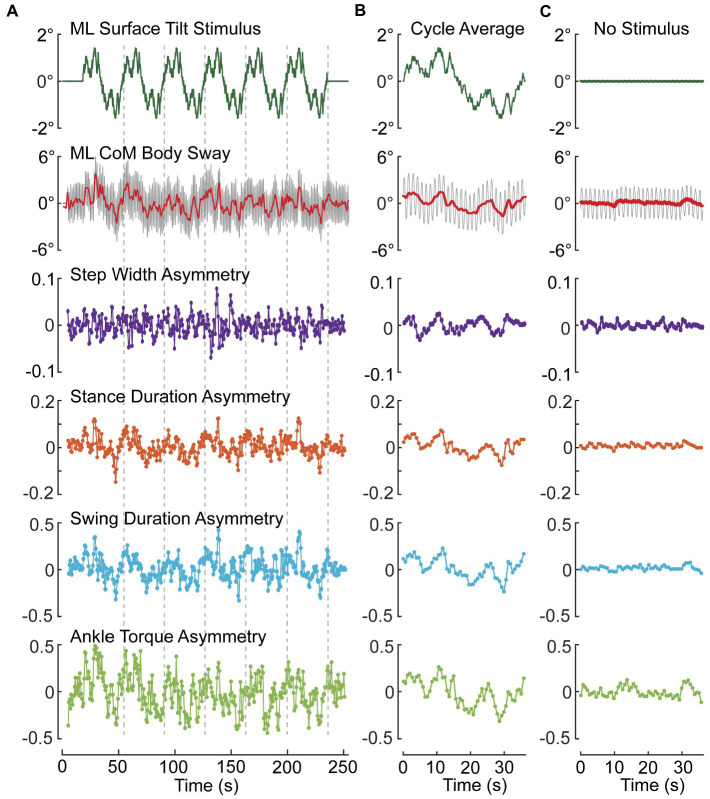
Example data from one subject showing modulation of ML CoM sway and asymmetry measures during an eyes-closed stepping-in-place trial on a tilting surface that rotated laterally during application of six cycles of a pseudorandom stimulus with 3° peak-to-peak amplitude **(A,B)** and average results from an eyes closed trial on a fixed, unmoving surface **(C)**. **(A)** Shows the entire time series of stimulus, path-corrected center-of-mass (CoM) sway and the four asymmetry measures. **(B)** Shows measures averaged over the last five 36.3-s duration cycles of the pseudorandom stimulus. **(C)** Shows cycle-averaged results for an eyes-closed stepping-in-place trial on a fixed surface demonstrating the lack of modulation of asymmetry measures compared to perturbed conditions. Stepping during both test conditions was metronome paced to give 56 steps per 36.3 s pseudorandom cycle (92.56 beats/min). The red line through the center of oscillating CoM motion (gray) is the 0.5-Hz lowpass filtered CoM sway.

Example results are shown from a subject performing metronome-paced SiP with eyes closed on a stance surface that tilted side-to-side during 6 cycles of a PRTS stimulus with 3° peak-to-peak amplitude (Figures 6A,B) and during an eyes closed SiP trial with no surface-tilt stimulus (Figure 6C). Similar to results from stance control tests (67), subjects tend to adjust their body orientation away from upright and towards alignment with the tilting surface. Deviation of the mean body orientation away from upright during SiP adds a bias to the destabilizing force due to gravity such that the mechanisms involved in maintaining dynamic stability must make appropriate adjustments. These adjustments are represented by the modulation of the four asymmetry measures that we have defined.

To the extent that a subject makes use of a particular mechanism, we expect the asymmetry measure associated with that mechanism will be modulated in relation to the deviation of body orientation from upright. Since all of the asymmetry measures were defined such that a positive (negative) asymmetry value is associated with a step-to-step action appropriate to correct for a rightward (leftward) bias in body orientation and motion, then we expect to see positive and negative modulation of the asymmetry values as the surface-tilt stimulus evokes rightward and leftward body leans away from upright. The step-to-step asymmetry measures in Figure 6A across the ~ 250-s duration of the SiP trials showed considerable variation making it difficult to appreciate the relation of the asymmetry measures to deviation in CoM sway (ΔCoM) from upright represented by the solid red line through the oscillating SiP sway. Averaging the stimulus, CoM sway, and asymmetry measures across the last 5 cycles of the PRTS stimulus reveals clear qualitative relationships between ΔCoM and all four asymmetry measures showing a continuous modulation in relation to the stimulus-evoked CoM sway.

When there was no surface-tilt stimulus, on average the body sway oscillations occurred about an upright orientation and there were minimal average deviations of CoM body orientation from 0° in comparison to the PRTS stimulus trial (Figure 6C vs. Figure 6B). Nevertheless, the average sway showed some small deviations from 0° and there was corresponding modulation in the asymmetry measures that were most evident in *ATA* and *SwDA*.

### 3.2. Regression analysis example

**Figure 7:**
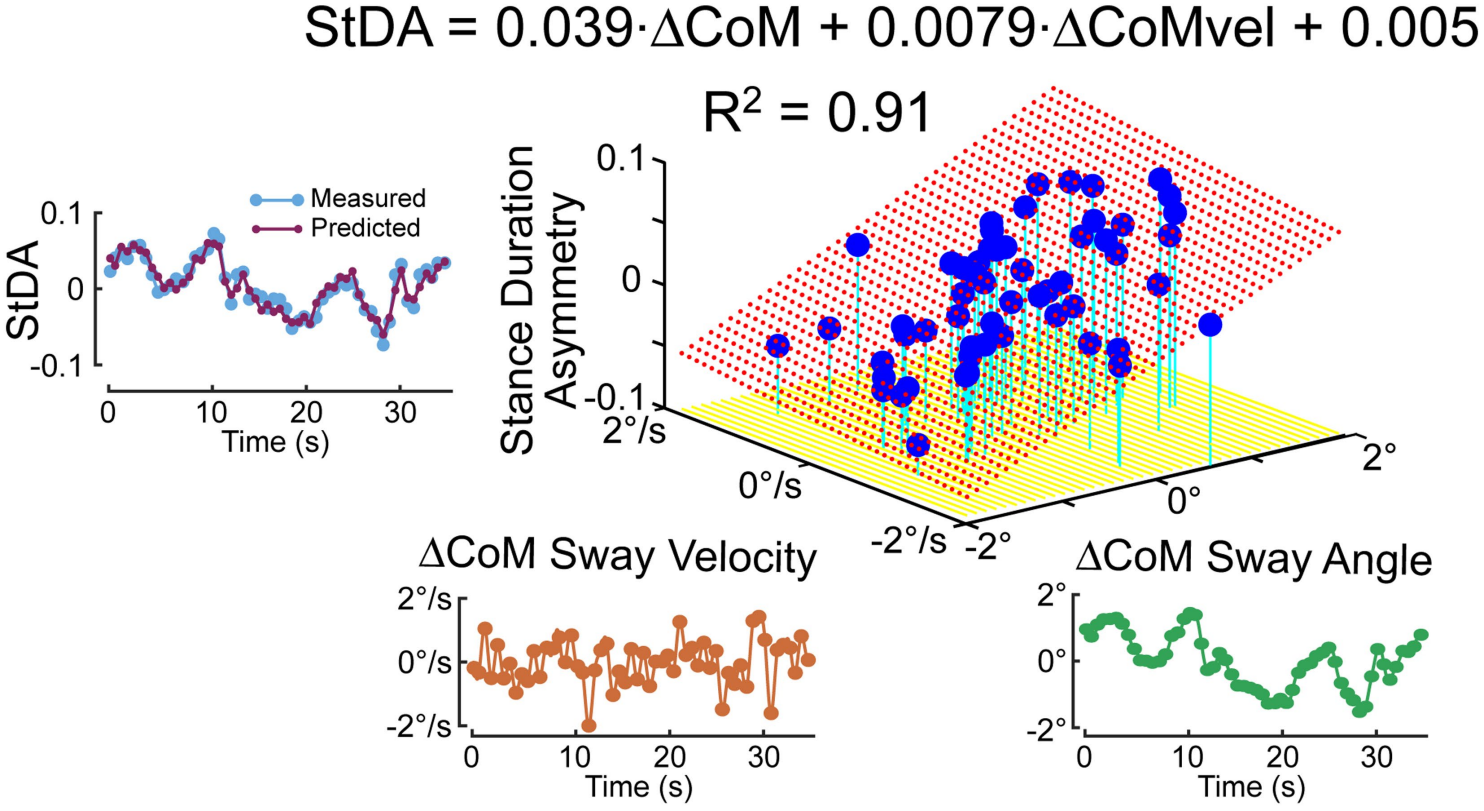
Example of regression analysis relating cycle-averaged Stance Duration Asymmetry (*StDA*) measures to average ΔCoM sway angle and ΔCoM angular velocity at time points corresponding to the mean times of occurrence of the *StDA* measures across the 56 time points 36.3-s duration of the pseudorandom PRTS surface tilt stimulus that perturbed ML balance during a stepping-in-place trial. The left plot shows that the measured cycle-averaged *StDA* is reliably predicted by results from the regression analysis. Red dotted surface is the regression fit.

To quantify the relationship between the stimulus-induced deviation of CoM orientation and the asymmetry measures, a regression analysis was performed that characterizes the extent to which a cycle-averaged asymmetry measure could be predicted based on the cycle-averaged ΔCoM orientation angles and angular velocity measures derived from the 0.5 Hz phaseless low-pass filtering of the recorded CoM sway angle. Figure 7 shows an example *StDA* regression analysis for one subject on one trial (same subject and trial as in Figure 6) demonstrating that a large proportion of the variance in the data was accounted for by the regression and consistent with an accurate prediction of the cycle-averaged *StDA* measures based on the regression equation.

**Figure 8:**
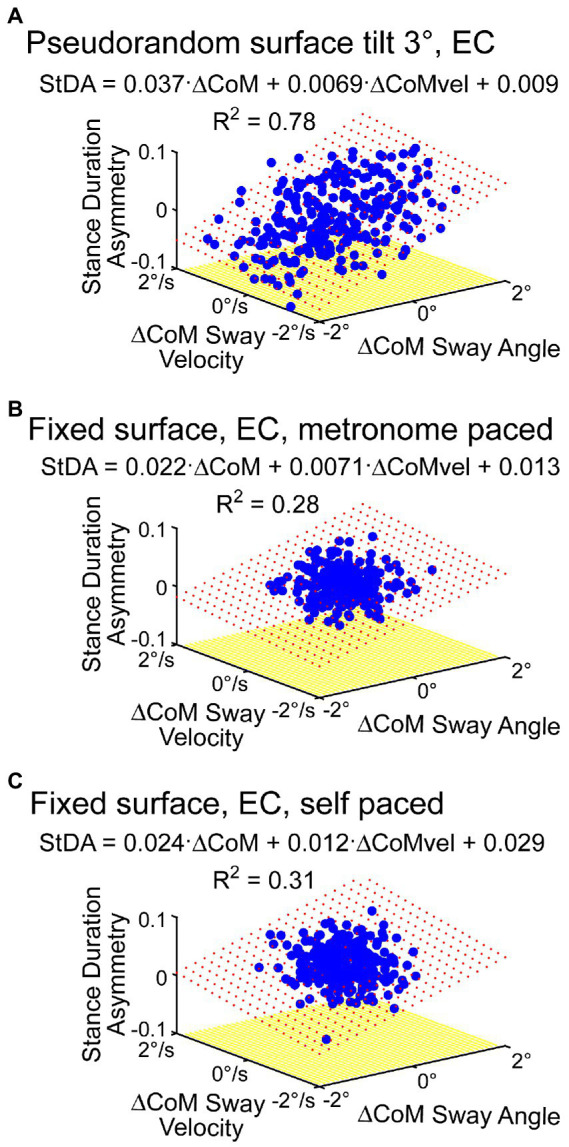
Examples of regression analysis applied to metronome-paced **(A,B)** and non-metronome-paced trials **(C)** using time series of individual asymmetry measures. **(A)** Shows regression analysis results of *StDA* using data from the same subject and trial as shown in Figure 7. **(B)** Shows *StDA* regression analysis results from a metronome-paced eyes-closed stepping-in-place trial performed on a fixed, unmoving surface. **(C)** Shows results from the same subject and same conditions as in **(B)** except with self-pacing rather than metronome pacing. Red dotted surface represents the regression fit.

While cycle averaging reduces variability and reveals average behavior, the individual step-to-step asymmetry measures can also be regressed against their associated ΔCoM orientation angles and angular velocity measures. The regression results shown in Figure 8A, using the same data set as in Figure 7 analysis, show similar regression coefficients. Additionally, the individual regression results could potentially reveal more detailed step-to-step behaviors. Although all four of the asymmetry measures are consistent with step changes that compensate for deviations from desired motion, corresponding changes may not occur in all measures on a given step. For example, in Figure 6A near the 220-s time point, the subject’s sway deviated to the right and was accompanied by positive deviations in *StDA* and *SwDA* but no clear changes in *SWA* and *ATA* were evident.

The results in Figure 6C showed some modulation in asymmetry measures in a no-stimulus condition even after averaging across cycles suggesting that there is sufficient step-to-step variability to estimate regression coefficient without the need to deliberately perturb balance. Results in Figure 8B support that view. Additionally, results demonstrated so far have been from metronome-paced trials where the subject maintained accurate pacing throughout the trials. Figure 8C shows regression results from the same subject from a self-paced zero-stimulus trial that shows similar regression coefficients to those obtained in the metronome-paced trial.

### 3.3. Effects of CoM cutoff frequency on regression analysis

A 0.5 Hz cutoff frequency was used to separate changes in CoM orientation from the dynamic oscillation that occurs during SiP under the assumption that the lower frequency changes in body orientation are the main determinates of step-to-step modulation of mechanisms controlling dynamic stability. But this choice was a heuristic one. To investigate the consequences of this choice of cutoff frequency, we took advantage of the odd symmetric properties of the PRTS stimulus (the second half of a PRTS cycle is the inverse of the first half) and that the metronome pacing was an even symmetric signal (within a PRTS cycle the second half of the metronome signal was the same as the first half). For subjects who stayed in phase with the metronome pacing, these signal properties allowed for a separation of the PRTS evoked response from the dynamic oscillation without the use of filtering.

**Figure 9:**
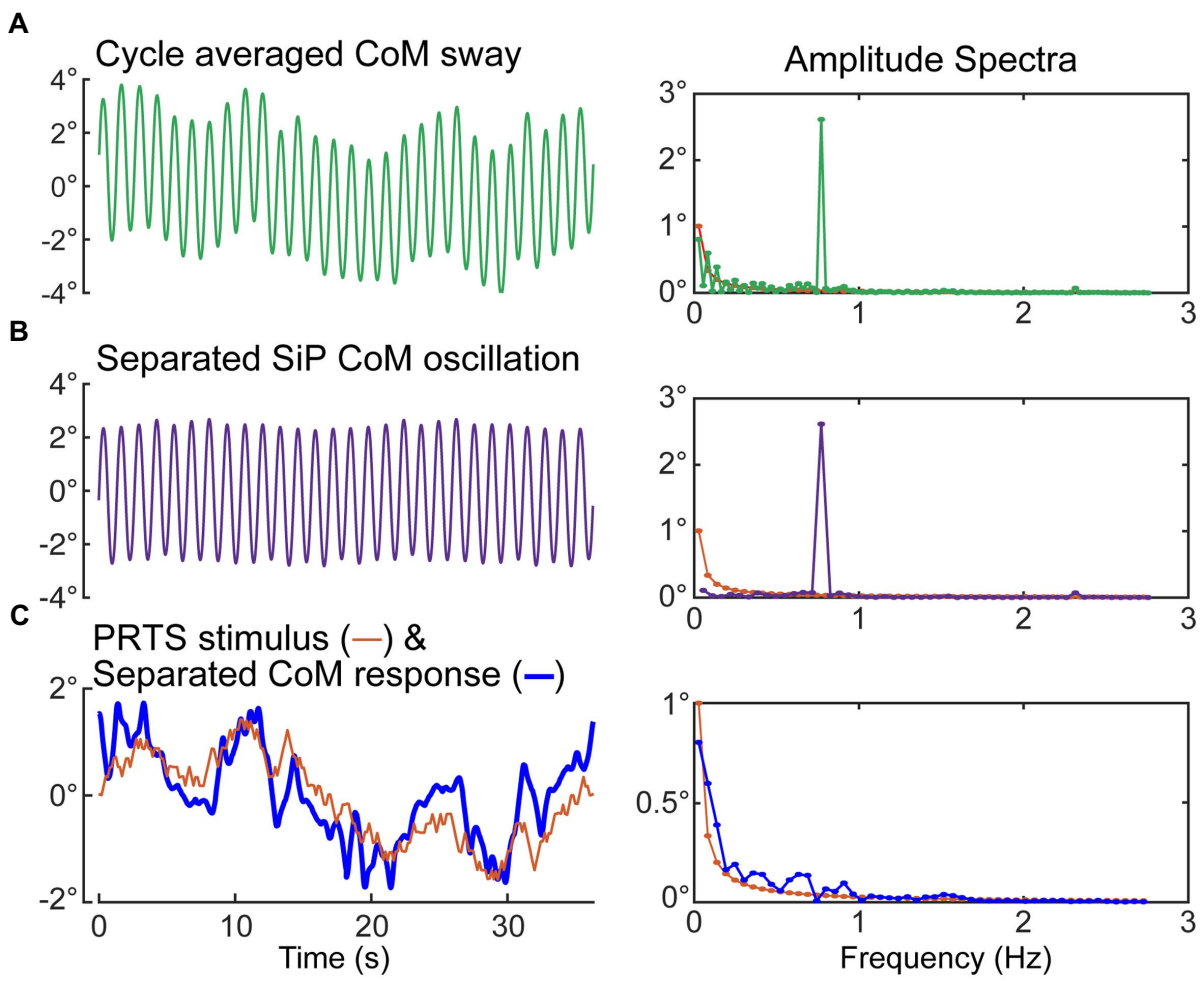
Separation of medial–lateral (ML) oscillation of the CoM during SiP from the CoM sway evoked by the ML pseudorandom PRTS surface-tilt stimulus. Metronome-paced trials with an even number of steps per stimulus cycle can be separated into the stimulus-evoked sway component and the oscillatory sway component. Left side plots show the cycle-average sway **(A)**, the separated oscillatory component at the stepping frequency **(B)**, and the separated stimulus-evoked CoM sway and the PRTS time course **(C)**. Corresponding amplitude spectra are shown in the right-side plots. The amplitude spectrum of the stimulus is shown in all right-side plots with only those harmonic components of the stimulus that have non-zero stimulus energy shown.

Specifically, subtracting the second half of the recorded CoM sway from the first half, and dividing by two, cancels out the dynamic oscillation and leaves the sway response to the PRTS. Then adding the second half of a PRTS-length cycle of the recorded CoM to the first half, and dividing by two, cancels the sway response to the PRTS stimulus and leaves the dynamic oscillation. Figure 9 shows an example of the separation process applied to the sway data shown in Figure 6A.

The separated PRTS CoM response waveform was used to reconstruct a full cycle of the PRTS CoM response and this full cycle was repeated to produce a 6 cycle CoM response to the PRTS. Finally, this 6-cycle CoM signal was processed using different cutoff frequencies and analyzed to calculate regression coefficients for the asymmetry measures to determine how varying the cutoff frequency affects these coefficients. In this analysis the asymmetry time points determined in the original analysis were used to sample the reconstructed CoM response angle and angular velocity waveforms that were used in the calculation of regression factors.

**Figure 10:**
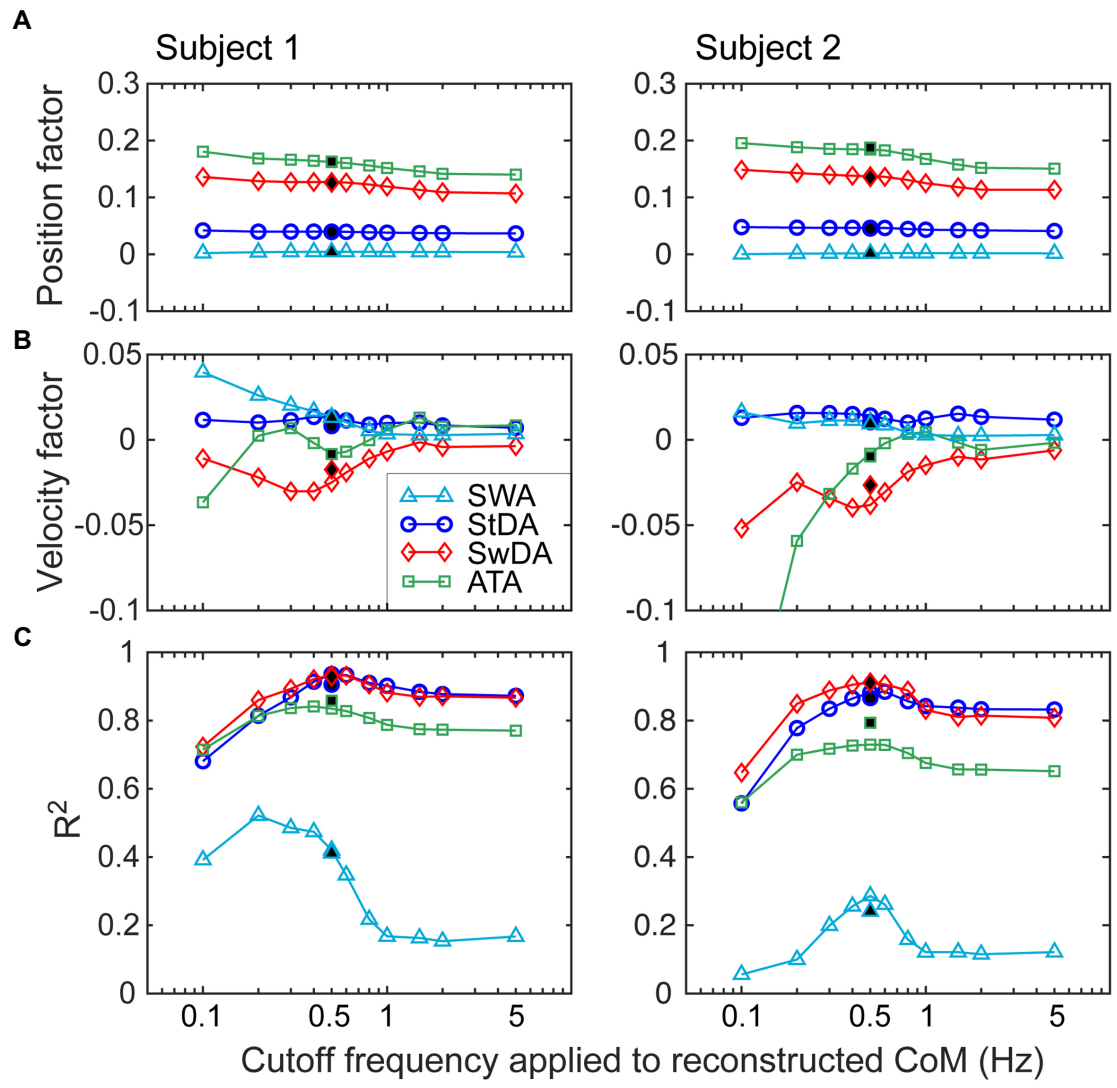
Investigation of the effects of the choice of the cutoff frequency of a lowpass filter applied to CoM stimulus-evoked motion on the calculation of position **(A)** and velocity **(B)** regression factors that relate CoM motion to asymmetry measures. Data from two different subjects are shown from eyes-closed, metronome-paced tests with 3° peak-to-peak surface-tilt stimuli. Lowpass filtering with cutoff frequencies ranging from 0.1 to 5 Hz was applied to the separated stimulus-evoked CoM sway (see Figure 9) before performing regression analysis to calculate CoM angular position and velocity factors. **(C)** *R*^2^ values indicating the variance accounted for by the regression analysis varied with the cutoff frequency. Filled points at 0.5 Hz are from the normal analysis that did not use the separated stimulus-evoked CoM sway.

Figure 10 shows examples from two subjects illustrating how regression analysis position and velocity factors changed as the cutoff frequency applied to the reconstructed CoM was varied from 0.1 to 5 Hz. The position and velocity factors for all four asymmetry measures were essentially unchanged for cutoff frequencies greater than about 1 Hz leading to a possible interpretation that the factors measured in this region provide unbiased measures of the position and velocity factors that relate stimulus-evoked sway to asymmetry changes. However, these results can only be obtained under conditions of metronome-paced stepping with specific properties of the perturbing stimulus (odd symmetry) in relation to the metronome signal (even symmetry). The example results in Figure 10 demonstrate that the potential for bias in regression factor measures exists when lower cutoff frequencies are used. Specifically, as the cutoff frequency was lowered the position factors remained relatively stable (constant for *SWA* and *StDA* and slightly increasing for *ATA* and *SwDA*). Velocity factors showed larger changes and the pattern of changes differed in the two examples. The filled symbols at 0.5 Hz show the values of the regression factors as determine by the original analysis (without reconstruction). The 0.5 Hz cutoff frequency was selected to be below the 0.77 Hz stepping frequency to eliminate most of the ML CoM stepping oscillation while preserving most of the stimulus-evoked CoM sway. Of note is that the variance accounted for by the CoM position and velocity fits to the asymmetry data tended to peak at cutoff frequencies just below the stepping frequency suggesting that there was a precision/accuracy tradeoff in estimating regression factors. That is, the higher *R*^2^ values suggest regression factor estimates would have lower variance but with possible biases that made them less accurate.

### 3.4. Asymmetry variability relationships with conventional gait variability measures

Gait variability, quantified by calculation of coefficient of variation (CV) of step width and gait timing measures, has been used as an indicator of the quality of balance control during gait with larger CVs indicative of reduced balance control and greater likelihood of falls in older individuals and those with neurological deficits (68–72). Step-to-step variability was also present in our asymmetry measures obtained in conditions with no applied stimulus. The extent to which conventional CV measures are correlated with step-to-step variability in our asymmetry measures could indicate that future studies using asymmetry variability measures could also identify gait disorders indicative of fall propensity and neurological decline.

Step-to-step variability in our asymmetry measures, quantified by calculating the SD of the asymmetry measures, was observed in conditions of both metronome-paced and non-metronome-paced conditions when no perturbing stimulus was presented. Supplementary Figure S2 illustrates results from non-metronome-paced SiP performed with eyes open and closed showing that CV measures of gait variability were well correlated with variability measures based on the SD of *StDA*, *SwDA*, and *SWA* values across all step cycles. Data used in Supplementary Figure S2 are given in Supplementary materials in addition to showing data for *ATA* SD measures (Supplementary Tables S1–S4). CV measures based on ankle torque measures are not practical since mean ankle torque values can be close to zero. Tables 1, 2 show results from comparisons between young and old subjects for the various asymmetry SD and conventional CV measures of variability. In eyes open conditions (Table 1) only the *ATA* SD measures showed significant differences between young and old subjects. In eyes closed conditions (Table 2) three of the four asymmetry SD measures showed significant differences between young and old while one of three CV measures was significant.

**Table 1:** Comparison of standard deviation (SD) of asymmetry measures and coefficient of variation (CV) of gait measures in young versus old subjects on self-paced and non-perturbed stepping-in-place tests performed with eyes open.

	Young mean (SD)	Old mean (SD)	*t* Test value	*p* Value
SWA SD	0.0172 (0.0038)	0.0178 (0.00326)	−0.532	0.598
Step Width CV	0.0503 (0.0189)	0.0468 (0.00940)	0.741	0.463
StDA SD	0.0188 (0.00402)	0.0202 (0.00333)	−1.17	0.249
Stance CV	0.0424 (0.0120)	0.0411 (0.00726)	0.428	0.671
SwDA SD	0.0599 (0.0202)	0.0635 (0.0160)	−0.618	0.540
Swing CV	0.119 (0.0530)	0.119 (0.0345)	0.041	0.968
ATA SD	0.0876 (0.0248)	0.123 (0.0722)	−2.08	**0.044**

SWA: Step Width Asymmetry, StDA: Stance Duration Asymmetry, SwDA: Swing Duration Asymmetry, ATA: Ankle Torque Asymmetry. Measures with bolded *p* values are significant at *p* < 0.05.

**Table 2:** Comparison of standard deviation (SD) of asymmetry measures and coefficient of variation (CV) of gait measures in young versus old subjects on self-paced and non-perturbed stepping-in-place tests performed with eyes closed.

	Young mean (SD)	Old mean (SD)	*t* Test value	*p* Value
SWA SD	0.0202 (0.00538)	0.021 (0.00409)	−0.871	0.389
Step width CV	0.0537 (0.0126)	0.0543 (0.00931)	−0.161	0.873
StDA SD	0.0239 (0.00469)	0.0322 (0.0131)	−2.66	**0.012**
Stance CV	0.0478 (0.00884)	0.0521 (0.0139)	−1.18	0.245
SwDA SD	0.0693 (0.0161)	0.0990 (0.0315)	−3.74	**<0.0001**
Swing CV	0.123 (0.0333)	0.153 (0.0466)	−2.36	**0.024**
ATA SD	0.130 (0.0398)	0.193 (0.112)	−2.38	**0.022**

SWA: Step Width Asymmetry, StDA: Stance Duration Asymmetry, SwDA: Swing Duration Asymmetry, ATA: Ankle Torque Asymmetry. Measures with bolded *p* values are significant at *p* < 0.05.

## 4. Discussion

The main contribution of this paper was to define and demonstrate the potential utility of new asymmetry measures for quantifying the step-to-step changes that are contributing to dynamic balance control. In experiments designed to investigate sensory integration during SiP gait we observed systematic deviations of CoM ML body orientation from earth vertical during SiP on a surface that continuously rotated side-to-side or while viewing a visual surround that rotated side-to-side. We noted that changes in ML body orientation were accompanied by modulations in step width, ankle torque, and in the duration of stance and swing phases in a manner consistent with their contributing to the maintenance of dynamic stability. We formulated four asymmetry measures from these gait parameters such that the positive or negative sign of all measures indicated the direction of corrective action afforded by the step-to-step changes in each gait parameter. We demonstrated that regression analysis could relate the change in an asymmetry measure to a deviation of ML CoM angular displacement and velocity.

We additionally demonstrated that the asymmetry measures could be used to quantify step-to-step changes in trials with no perturbing stimulus. We showed that the variability of our asymmetry measures was correlated with conventional measures of gait variability but may possibly have advantages over conventional measures in that they (1) relate directly to control mechanisms; and (2) they were better able to identify differences between younger and older healthy adults.

### 4.1. Control mechanisms

For control based on foot placement, if there was a rightward bias in body orientation and if the next step was with the right foot then placing that foot further to the right would result in the CoM being further to the left of the right foot position during the right leg stance phase resulting in greater gravity induced torque toward the left that corrects for the rightward bias in body orientation. If the next step is with the left foot, then a more medial placement of that foot would reduce the magnitude of the rightward gravity induced torque and would also contribute to correcting for a rightward bias in body orientation. The regulation of step placement is understood to be an important contributor to dynamic balance control during gait (5, 7, 41, 45, 48, 73). Figure 6 shows a change in *SWA* in response to ML body orientation deviations produced by the PRTS perturbation where the cycle-averaged *SWA* magnitude moved complementary with the cycle-averaged ΔCoM body sway. For example, at the time point around 30 s the ΔCoM body sway had a negative amplitude (demonstrating an average tilt of the body to the left) and the SWA at that time point showed a corresponding negative amplitude consistent with a change in step width that provided compensation for the stimulus-evoked leftward ML body tilt.

For dynamic balance control based on ankle torque, an ankle inversion of the right foot and eversion of the left foot during stance phases would generate a corrective torque the compensates for a rightward bias in body orientation. Contribution of ankle torque is recognized as a contributor to dynamic balance control in the frontal plane (8, 34). Figure 6 shows a change in cycle-averaged *ATA* in response to PRTS perturbation-induced body tilt whose amplitude was in a similar direction to the cycle-averaged ΔCoM body sway response to the perturbation consistent with a step-to-step change in ankle torque that compensated for the stimulus-evoked body lean.

Less well appreciated is the potential for step timing regulation to contribute to dynamic balance control. Studies of quadrupedal robots have demonstrated that ML balance during gait can be achieved entirely by regulating step timing regulated by limb loading and unloading (43, 44). Step timing changes have been noted in experimental studies in cats and humans (21, 40, 41). Control can be achieved by regulating the duration of the stance phase and/or the swing phase of gait. For a rightward bias in body orientation increasing the duration of the right leg stance phase will lengthen the time over which a gravity induced torque toward the left is present. Reducing the duration of the left leg stance phase will shorten the time over which a rightward gravity torque is present. Both of these actions would compensate for a rightward bias in body orientation. Figure 6 shows a change in *StDA* in response to the PRTS perturbation-induced body tilt demonstrating this corrective action. For example, around time 10 s the cycle-averaged ΔCoM body sway had a positive deviation (indicating body tilt toward the right) and the *StDA* had a corresponding positive amplitude indicating a longer right leg stance duration compared to the left. The longer right leg stance duration provided a longer duration over which a leftward directed gravity torque could act to compensate for the rightward lean.

A shorter right leg swing duration would be consistent with either extending the duration of the previous right leg stance phase or ensuring an earlier foot contact time on the next right leg stance phase. Both scenarios would extend the time over which a leftward gravity induced torque was present during right leg stance. Lengthening the left leg swing duration would similarly contribute to reducing the left leg stance phase. Thus, a combination of a shorter right leg and longer left leg swing phases would contribute to compensation for a rightward biased body orientation during gait. Figure 6 shows modulation of cycle-averaged *SwDA* consistent with changes in swing duration that contributed to compensation for changes in the stimulus-evoked ΔCoM.

One might consider that metronome paced stepping would result in a perfect correlation between *StDA* and *SwDA* measures such that they would be completely redundant. But even if a subject’s heel strikes were perfectly synchronized with the metronome, different segments of the stepping cycle contribute to the *StDA* and *SwDA* calculations that correspond to approximately the same time point. For example, times *t_StDA2_* in Figure 4 and *t_SwDA1_* in Figure 5 both occur at approximately the middle of the double support phase near the beginning of the second right leg stance segment. The *StDA_2_* value depends on the duration of the second right and the first left leg stance durations (*StD_3_* and *StD_2_*). But, assuming heel strike synchronization with the metronome, the *SwDA_1_* value depends on the swing duration associated with the first right leg gait segment (i.e., *SwD_1_* which depends on the first right leg stance duration *StD_1_*) and the first left leg gait segment (i.e., *SwD_2_* which depends on the first left leg stance duration *StD_2_*). Thus, the *StDA* and *SwDA* measures will not be fully redundant since they are a function of different gait segments. Additionally, the possibility exists that stance and swing duration could be driven by separate control mechanisms particularly under conditions where there is no metronome pacing constraining segments of the gait cycle.

### 4.2. Asymmetry measures

The above descriptions of the four dynamic balance control mechanisms we considered emphasize the complementary nature of actions of the two legs which suggested that asymmetry measures which compared metrics from two adjacent steps could provide useful quantification of the contribution of these mechanisms to dynamic balance control. Various asymmetry measures are widely employed in gait studies (74–77). A common measure, referred to as a symmetry index (SI) measure (78), compares the difference between two gait measures to their mean value. Our *SWA*, *StDA*, and *SwDA* measures are of this form except that we chose to divide the difference by the sum rather than the mean since this affords some desirable features (74). Our *ATA* measure appears to differ from this form since the numerator is the sum of two measures proportional to ankle torque. However, while the two numerator terms have the same sign, one represents an eversion torque and the other an inversion torque. Therefore, in terms of ankle-referenced torque measures their signs are opposite.

While our asymmetry measures could have been calculated without regard to their having any relationship to body motion, our calculations were formulated with the desired property that the sign of the asymmetry measures would be indicative of step-to-step changes that compensated for deviations in ML CoM body orientation during gait. With the exception of the *ATA* calculation, it was necessary to consider which foot made the first step. With our definitions all of the asymmetry measures had positive (negative) signs when the balance control mechanisms they represented were compatible with generating corrective actions that compensated for rightward (leftward) deviations of ML body orientation during gait.

It is important to point out that the asymmetry measures do not provide a measure of the magnitude of the corrective action (torque) because (1) they are normalized measures and (2) they are based on measures associated with a single point in time with that time being the average of two discrete time points. However, for a particular asymmetry measure, a larger value indicates that a greater balance correction was made by the mechanism that that asymmetry measure represents. We consider that the regression analysis will be most useful in providing insight into dynamic balance control variations among people, between different subject groups, and as a function of environmental conditions. For example, constraining changes in step width or the ability to generate ankle torque would be expected to result in increased contributions from mechanisms that were not constrained (33, 73). The increased contributions would be represented by larger regression coefficient values that relate CoM motion to asymmetry measures for those measures that substituted for the constrained mechanisms.

The example data shown in Figure 6 from a metronome-paced trial demonstrated that an ML surface-tilt perturbation evoked deviation from an upright orientation during SiP. All four asymmetry measures demonstrated that step width, stance duration, swing duration, and ankle torque mechanisms were all contributing to dynamic balance control. Furthermore, the cyclic nature of the applied pseudorandom stimuli allowed for across-cycle averaging of CoM sway and asymmetry measures (Figure 6B). These averaged results could be further analyzed to calculate regression factors relating CoM sway angle and angular velocity to the asymmetry measure (Figure 7) to give insight into what dynamic aspects of CoM sway (i.e., position and/or velocity sensitivity) were associated with changes in a balance control mechanism. The application of cyclic stimuli to evoke changes in body orientation had the advantage that the analysis could average measures across cycles to reduce variability. However, the similar regression results obtained without averaging (Figure 8A) suggested that reliable results could be obtained without averaging.

Cycle averaging and CoM regression analysis can also be applied to data obtained in non-metronome paced trials when care is taken to select an appropriate lowpass cutoff frequency for filtering the CoM signal but with the recognition that the cutoff frequency has some effect on the calculated asymmetry values (Figure 10).

Additionally, with metronome pacing with an even integer number of step cycles per PRTS stimulus cycle (or other stimuli with odd-harmonic properties where the second half of the stimulus is the inverse of the first half), it is possible to separate the stimulus-evoked sway from the oscillatory component at the stepping frequency if the subject is able to keep pace with the metronome (Figure 9). This separation would allow a detailed investigation of the stimulus-evoked sway at frequencies both below and above the stepping frequency. Such an analysis would not be possible with non-metronome paced tests.

### 4.3. Asymmetry variability in non-perturbed conditions

Good correlations between the SD of asymmetry and conventional CV measures were observed during unperturbed SiP (Supplementary Figure S2). If variability measures during walking show similar correlations between CV measures and asymmetry SD measures, then asymmetry SD measures could provide an equally informative indicator of fall risk and balance degradation due to aging and neurological deficits as demonstrated in numerous studies (24, 68–72, 79–83). If asymmetry SD measures during SiP were correlated with CV measures during walking gait, then this could support using a SiP protocol to evaluate fall risk and balance degradation since a SiP paradigm is relatively simple to implement.

The demonstration that our asymmetry measures were modulated by CoM motion in both perturbed and unperturbed SiP (Figures 6–8) provides evidence that these asymmetry measures are likely indicative of the contribution of various mechanisms to balance control during gait. Thus, covariation of conventional CV and asymmetry SD measures could provide a bridge that links CV measures to specific balance control mechanisms rather than just being an indicator of the quality of balance control. In this way our asymmetry analyses may provide additional insights into what specific deficits in balance control mechanisms are producing increased gait variability.

By analogy to standing balance control, body sway variability can arise from sensory, motor, and central processing noise sources but is also influenced by the dynamic properties of the overall control system (84, 85). While spontaneous sway variability during stance provides some indication of overall balance system behavior, the application of external perturbations and the use of system identification methods that are appropriate for control systems where feedback plays a major role (86) can enhance our ability to identify which components of the system are responsible for balance deficits (87). While some aspects of standing balance control are likely relevant to gait, the existence of multiple mechanisms contributing to balance during gait, the necessary coordination between these mechanisms, and the shifting of control action from one leg to the other poses a challenge to fully account for balance control during gait. Additional considerations regarding the role of feedforward control, as used in visually-guided step placement (88), are needed for a more complete understanding of balance control during gait.

### 4.4. Limitations

In this primarily methods-based report we defined asymmetry measures to characterize the contributions of four different mechanisms to dynamic balance control in the frontal plane. The measures were developed to quantify results from SiP tests that were obtained in a group of 20 younger and 20 older adults tested under a variety of conditions. The limited example data we presented to illustrate our methods show representative results but final conclusions require publication of the full study which is in preparation.

The literature using a SiP paradigm to investigate frontal plane balance control is limited but suggests there are similarities between SiP and forward walking gait (18–20). However, SiP is not walking and there are balance mechanisms that contribute to frontal plane balance control that involve forward walking [AP/ML crosstalk associated with push-off torque (4, 8, 35, 38) and steering control (39)]. Therefore, even if our asymmetry measures provide an excellent characterization of mechanisms contributing to frontal plane balance during SiP they will be incomplete as regards the full set of mechanisms contributing to frontal plane balance during walking. Additionally, a comparison of asymmetry measures from SiP and walking will be necessary to determine the extent to which SiP results are similar to or different from walking. A preliminary analysis of data we have collected during forward walking gait indicates that the methods developed for SiP analysis also apply to walking gait but a detailed comparison requires a full analysis.

Subjects performing SiP also needed to control their sagittal plane balance. But none of our asymmetry measures provided any information about sagittal plane balance control. The absence of forward motion during SiP reduces the likelihood that any characterization of sagittal plane balance during SiP would be relevant to mechanisms contributing to sagittal plane balance during walking.

We demonstrated that metronome pacing facilitates some types of analysis (Figures 6, 7, 9) but is an unnatural condition that may affect results. It is currently unknown the extent to which metronome pacing influences results from SiP tests.

### 4.5. Future directions

We suggest that use of a SiP protocol is under-explored and may be a useful paradigm for investigating the contributions of mechanisms that are known to be important contributors to frontal plane balance during gait (i.e., step width and ankle torque) and less well understood contributions from variations in step timing. The experimental equipment needed for SiP tests is less complex than needed for overground walking and for treadmill walking especially if perturbations such as tilting of the surface are found to be informative. SiP tests can readily be performed with eyes closed to provide a focus on the contributions of proprioceptive and vestibular cues for balance control. Anecdotal reports suggest that eyes closed walking is uncomfortable for subjects and not possible with treadmill walking. However, the utility of a SiP protocol needs to be demonstrated in comparison with better-known paradigms, such as treadmill walking, in experiments that similarly explore balance control mechanisms under a variety of conditions (varying pace, varying mean step width, application of perturbations such as visual motion and externally applied forces, galvanic vestibular stimulation). If SiP results correspond well with results from walking gait, the SiP protocol could have an important future role in clinical evaluations.

We demonstrated good correlations between traditional variability measures and variability of our asymmetry measures from non-metronome paced SiP tests performed with no perturbation. Additionally, our asymmetry variability measures were better able to distinguish between younger and older adults than traditional measures (Tables 1, 2). Because traditional measures based on walking gait have previously been shown to relate to aging, falls, and various neurological disorders as described in the Introduction, it would be important to know if (1) quantification of walking gait variability using asymmetry measures rather than traditional measures would be better at distinguishing between groups; and (2) if asymmetry variability from SiP tests could be equally or possibly more effective in distinguishing between groups than variability measures from walking gait. The latter result would support the clinical use of SiP testing and quantification based on the variability of easily measured asymmetry variables to screen patients for gait-related balance deficits.

## Data availability statement

The original contributions presented in the study are included in the article/Supplementary material, further inquiries can be directed to the corresponding author/s.

## Author contributions

RP contributed the original conception and design of the study. RP, AG-F, and PH contributed to algorithm development. RP and AG-F contributed to collection, analysis, and interpretation of the data. RP wrote the first draft. All authors contributed to the article and approved the submitted version.

## Funding

The work was supported by the United States Department of Veterans Affairs, Veterans Health Administration, Rehabilitation Research and Development Service, Merit Award No. C1951-R/I01 RX001951 (to RP) and the National Institutes of Health, National Institute on Deafness and Other Communication Disorders T35 training grant DC008764 (supporting PH). Resources and facilities at the VA National Center for Rehabilitative Auditory Research (NCRAR) were supported by Award No. C2361/I50 RX002361 at the VA Portland Health Care System in Portland, Oregon. The contents do not represent the views of the United States Department of Veterans Affairs or the United States Government.

## Conflict of interest

The authors declare that the research was conducted in the absence of any commercial or financial relationships that could be construed as a potential conflict of interest.

## Publisher’s note

All claims expressed in this article are solely those of the authors and do not necessarily represent those of their affiliated organizations, or those of the publisher, the editors and the reviewers. Any product that may be evaluated in this article, or claim that may be made by its manufacturer, is not guaranteed or endorsed by the publisher.

## Footnotes

^1^https://ni.com

^2^https://www.transducertechniques.com/

^3^https://global.sharp/

^4^https://www.midoriamerica.com/

^5^https://www.cmccontrols.com/

^6^https://www.amacoil.com/

^7^https://www.aerotech.com/

^8^https://www.servodynamics.com/
