# Supplementary material for: Asymmetry measures for quantification of mechanisms contributing to dynamic stability during stepping-in-place gait
Source: Front Neurol. 2023 Apr 20;14:1145283. doi: 10.3389/fneur.2023.1145283 (PMC10157157; doi:10.3389/fneur.2023.1145283)
Supplement: Supplementary file 1 [file Data_Sheet_1.PDF]

Supplementary Material

Asymmetry Measures for Quantification of Mechanisms Contributing to Dynamic Stability during Stepping-in-Place Gait

Robert J. Peterka*, Apollonia Gruber-Fox, Paige K. Heeke

*** Correspondence:**
Robert J. Peterka: Robert.Peterka@va.gov

Supplementary materials include 1) Figure S1 showing the test device used to test dynamic balance using a stepping-in-place (SiP) protocol, 2) Tables listing SiP individual variability measures, and 3) Figure S2 showing comparisons between variability measures in young and old subjects.

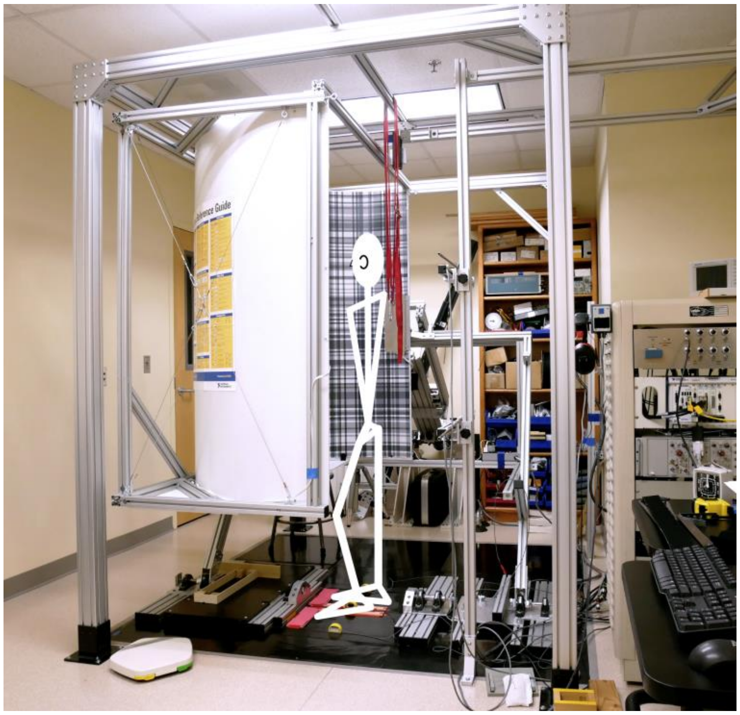

Supplementary Figure S1. Custom balance test device used for SiP experiments. A subject (white stick figure) stepped in place on a stance surface that could tilt side-to-side under servo-motor control about an axis at ankle joint height and passing between the subject's feet. On eyes open trials the subject viewed a visual scene with a complex high contrast random plaid pattern. A separate servo motor could also tilt the visual surround about an axis aligned with the stance surface rotation axis. Separate force plates under the two feet recorded vertical forces and center-of-pressure locations under the feet. Lateral body motions were measured at hip and shoulder levels. Thin foam pads in a T-formation on the stance surface provide tactile feedback that a subject could use to minimize drift in stepping location during SiP. Optical distance measurement devices placed on the outer edges and between the feet measured the location of the feet when they were on the surface.

Table S1. Standard deviation (SD) of asymmetry measures and coefficient of variation (CV) of gait measures in young adults (age range 25-43 years, mean 32 years) on self-paced and non-perturbed stepping-in-place tests performed with eyes open.

**Subject**	***SWA* SD**	**Step Width CV**	***StDA* SD**	**Stance CV**	***SwDA* SD**	**Swing CV**	***ATA* SD**
Y1	0.022	0.051	0.0161	0.0312	0.0557	0.1027	0.0679
Y2	0.0137	0.043	0.0147	0.0621	0.0844	0.2105	0.0882
Y3	0.0123	0.0378	0.0161	0.0287	0.0514	0.0841	0.0534
Y4	0.0177	0.0456	0.0185	0.0354	0.0537	0.1035	0.0653
Y5	0.0219	0.0425	0.0233	0.0416	0.0663	0.1131	0.0955
Y6	0.0223	0.0673	0.0188	0.0436	0.0404	0.0711	0.078
Y7	0.0193	0.0426	0.0229	0.0461	0.0511	0.0959	0.0906
Y8	0.0185	0.0489	0.0221	0.0483	0.0683	0.1178	0.1181
Y9	0.0164	0.04	0.0198	0.0365	0.0676	0.1148	0.094
Y10	0.0196	0.0411	0.0218	0.0709	0.0942	0.242	0.1048
Y11	0.0163	0.0705	0.0218	0.0474	0.0731	0.109	0.0908
Y12	0.0175	0.0383	0.0159	0.0322	0.0353	0.0628	0.1406
Y13	0.0097	0.0372	0.0122	0.0313	0.0478	0.1238	0.0427
Y14	0.0162	0.0731	0.0196	0.0449	0.0521	0.1113	0.0812
Y15	0.0153	0.0396	0.012	0.0225	0.0249	0.0511	0.097
Y16	0.0099	0.0222	0.0183	0.0348	0.0551	0.0935	0.0631
Y17	0.0194	0.0567	0.0222	0.0465	0.0575	0.0963	0.0668
Y18	0.0176	0.058	0.0131	0.0357	0.033	0.0754	0.107
Y19	0.0229	0.1105	0.0266	0.0511	0.0937	0.1861	0.1291
Y20	0.0156	0.0406	0.0211	0.0574	0.093	0.2173	0.0782
**Mean**	0.0172	0.0503	0.0188	0.0424	0.0599	0.119	0.0876
**SD**	0.0038	0.0189	0.00402	0.0120	0.0202	0.0530	0.0249

SWA: Step Width Asymmetry, *StDA*: Stance Duration Asymmetry, *SwDA*: Swing Duration Asymmetry, *ATA*: Ankle Torque Asymmetry.

Table S2. Standard deviation (SD) of asymmetry measures and coefficient of variation (CV) of gait measures in young adults (age range 25-43 years, mean 32 years) on self-paced and non-perturbed stepping-in-place tests performed with eyes closed.

**Subject**	***SWA* SD**	**Step Width CV**	***StDA* SD**	**Stance CV**	***SwDA* SD**	**Swing CV**	***ATA* SD**
Y1	0.0193	0.0463	0.0206	0.0456	0.0673	0.1415	0.1083
Y2	0.0158	0.0604	0.0181	0.0544	0.0822	0.1764	0.1342
Y3	0.0182	0.047	0.022	0.0383	0.0687	0.1094	0.0822
Y4	0.0205	0.0489	0.021	0.0372	0.0649	0.0969	0.0878
Y5	0.0208	0.0475	0.0244	0.042	0.0555	0.0932	0.1727
Y6	0.0188	0.0652	0.0247	0.048	0.0575	0.0981	0.0902
Y7	0.0189	0.0437	0.0261	0.0437	0.0631	0.0929	0.1308
Y8	0.021	0.0507	0.0339	0.0624	0.0799	0.1227	0.1647
Y9	0.0322	0.0659	0.0311	0.0457	0.0782	0.1	0.1823
Y10	0.0332	0.0937	0.0264	0.0591	0.0778	0.1383	0.2195
Y11	0.017	0.0499	0.0307	0.0507	0.0958	0.1485	0.0887
Y12	0.0139	0.0445	0.0199	0.0381	0.0638	0.1279	0.1154
Y13	0.0177	0.0505	0.0168	0.0365	0.0727	0.195	0.0869
Y14	0.018	0.0408	0.0224	0.055	0.0573	0.0962	0.1103
Y15	0.0182	0.0513	0.0167	0.0319	0.0308	0.0575	0.1422
Y16	0.0102	0.0462	0.0226	0.0499	0.0558	0.1168	0.1114
Y17	0.0205	0.0447	0.0269	0.0593	0.0857	0.1474	0.0959
Y18	0.0229	0.0514	0.021	0.0476	0.0541	0.1001	0.1441
Y19	0.0262	0.0738	0.0268	0.0605	0.1009	0.1621	0.193
Y20	0.0208	0.0514	0.0256	0.0501	0.0745	0.14	0.142
**Mean**	0.0202	0.0537	0.0239	0.0478	0.0693	0.123	0.130
**SD**	0.00538	0.0126	0.00469	0.00884	0.0161	0.0333	0.0398

SWA: Step Width Asymmetry, *StDA*: Stance Duration Asymmetry, *SwDA*: Swing Duration Asymmetry, *ATA*: Ankle Torque Asymmetry.

Table S3. Standard deviation (SD) of asymmetry measures and coefficient of variation (CV) of gait measures in older adults (age range 65-82 years, mean 72 years) on self-paced and non-perturbed stepping-in-place tests performed with eyes open.

Subject	*SWA* SD	Step Width CV	*StDA* SD	Stance CV	*SwDA* SD	Swing CV	*ATA* SD
O1	0.0191	0.0531	0.0203	0.0477	0.08	0.1436	0.4065
O2	0.0211	0.0525	0.0217	0.04	0.0535	0.08	0.134
O3	0.0156	0.0451	0.0172	0.0544	0.0478	0.1145	0.143
O4	0.0193	0.0439	0.023	0.0391	0.0543	0.0871	0.0976
O5	0.0254	0.05	0.0254	0.0509	0.0785	0.1474	0.1262
O6	0.0176	0.0591	0.0169	0.0299	0.045	0.0778	0.1567
O7	0.0177	0.0487	0.0269	0.0468	0.0884	0.1509	0.1219
O8	0.0112	0.0268	0.0171	0.0301	0.0646	0.1071	0.0995
O9	0.0197	0.0531	0.0242	0.0524	0.0733	0.1499	0.0597
O10	0.0176	0.036	0.0213	0.0439	0.0703	0.1047	0.0907
O11	0.0199	0.0555	0.0254	0.0458	0.0745	0.1185	0.1117
O12	0.02	0.053	0.0189	0.0359	0.0645	0.1207	0.0854
O13	0.0186	0.05	0.0185	0.038	0.0747	0.1543	0.1017
O14	0.0184	0.0659	0.0171	0.0352	0.0672	0.1422	0.0822
O15	0.0178	0.044	0.0219	0.0457	0.0535	0.1399	0.1353
O16	0.0127	0.0411	0.0165	0.041	0.0599	0.1546	0.1168
O17	0.0164	0.0494	0.0177	0.0352	0.0421	0.0683	0.1174
O18	0.0192	0.0387	0.0169	0.0306	0.0297	0.0438	0.1262
O19	0.0118	0.0338	0.0176	0.0389	0.0916	0.1681	0.0417
O20	0.0169	0.0367	0.0199	0.0401	0.0561	0.0972	0.1096
**Mean**	0.0178	0.0468	0.0202	0.0411	0.0635	0.119	0.123
**SD**	0.00326	0.00940	0.00333	0.00726	0.0160	0.0345	0.0722

SWA: Step Width Asymmetry, *StDA*: Stance Duration Asymmetry, *SwDA*: Swing Duration Asymmetry, *ATA*: Ankle Torque Asymmetry.

Table S4. Standard deviation (SD) of asymmetry measures and coefficient of variation (CV) of gait measures in older adults (age range 65-82 years, mean 72 years) on self-paced and non-perturbed stepping-in-place tests performed with eyes closed.

**Subject**	***SWA* SD**	**Step Width CV**	***StDA* SD**	**Stance CV**	***SwDA* SD**	**Swing CV**	***ATA* SD**
O1	0.0196	0.0661	0.0249	0.0481	0.0875	0.1338	0.1044
O2	0.0283	0.0581	0.0298	0.0479	0.1072	0.1581	0.5509
O3	0.0216	0.0545	0.026	0.0452	0.0755	0.1098	0.2296
O4	0.028	0.0644	0.0758	0.0789	0.1335	0.1804	0.3493
O5	0.0278	0.0637	0.0484	0.0794	0.1594	0.2419	0.3016
O6	0.0241	0.0439	0.0248	0.0395	0.0753	0.1233	0.2082
O7	0.0209	0.0577	0.051	0.0846	0.1672	0.2396	0.2353
O8	0.0208	0.046	0.0209	0.0404	0.0728	0.1499	0.1203
O9	0.0205	0.0451	0.0367	0.0584	0.1253	0.1986	0.0757
O10	0.0193	0.0573	0.0341	0.0553	0.0982	0.1451	0.1054
O11	0.0176	0.0653	0.0363	0.0575	0.1389	0.227	0.1479
O12	0.0221	0.0618	0.03	0.052	0.0991	0.1572	0.1111
O13	0.0211	0.0484	0.0305	0.0541	0.0918	0.127	0.1537
O14	0.0229	0.0603	0.0232	0.039	0.0831	0.1202	0.1379
O15	0.0195	0.0501	0.0258	0.048	0.0687	0.1215	0.1982
O16	0.0157	0.0589	0.0209	0.038	0.0896	0.1825	0.1567
O17	0.0264	0.056	0.0247	0.044	0.0571	0.0939	0.2034
O18	0.0232	0.0517	0.0269	0.041	0.0542	0.0722	0.2416
O19	0.0124	0.0282	0.0235	0.0413	0.1025	0.1555	0.0627
O20	0.0189	0.0477	0.0288	0.0502	0.0921	0.1273	0.1704
**Mean**	0.021	0.0543	0.0322	0.0521	0.0990	0.153	0.193
**SD**	0.00409	0.00931	0.0131	0.0139	0.0315	0.0466	0.112

SWA: Step Width Asymmetry, *StDA*: Stance Duration Asymmetry, *SwDA*: Swing Duration Asymmetry, *ATA*: Ankle Torque Asymmetry.

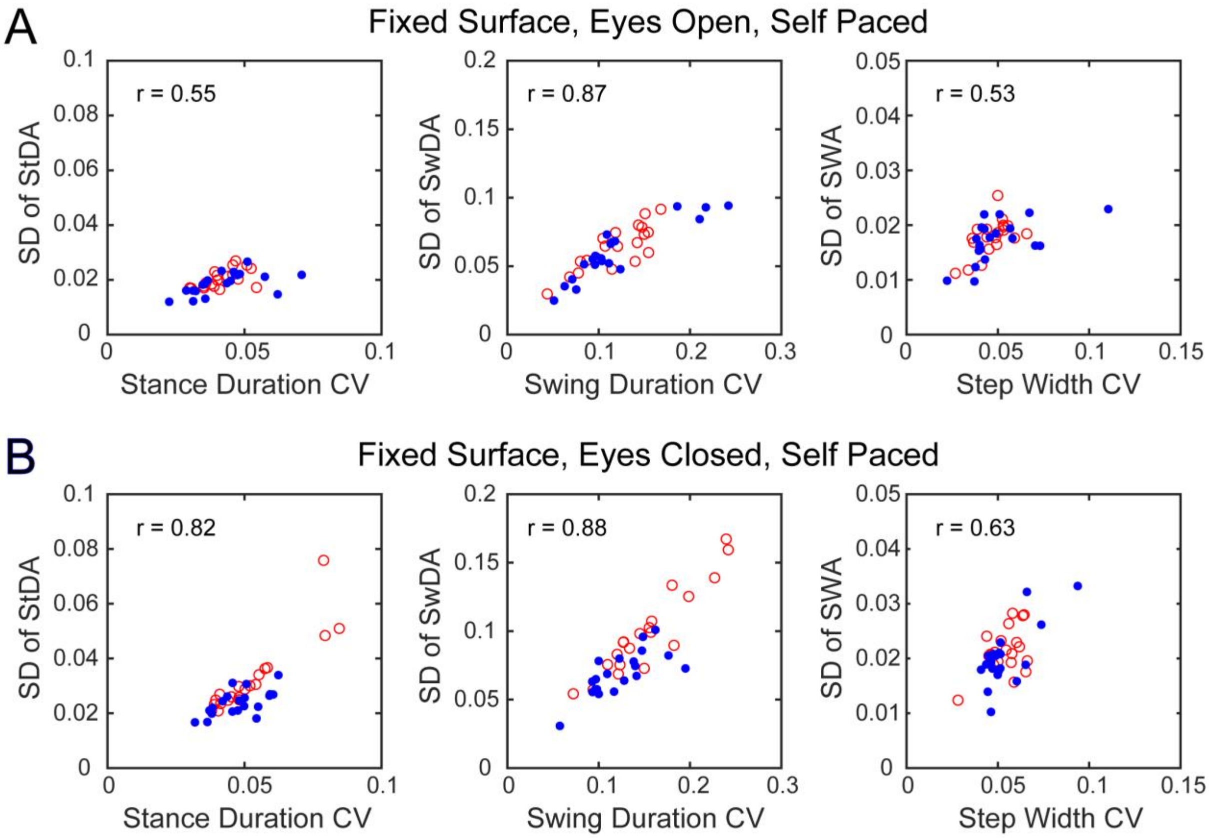

Supplementary Figure S2. Comparison of gait variability measures from conventional methods using the coefficient of variation (CV; standard deviation divided by the mean) of stance duration, swing duration, and step width to gait variability measures based on the standard deviation (SD) of *StDA*, *SwDA*, and *SWA* in eyes open conditions (A) and eyes closed conditions (B). Data are from 20 older subjects (open symbols) and 20 younger adults (filled symbols). Results are from non-metronome paced tests performed on a fixed stance surface.
